# How system justification sustains sexual inequality: The case of the orgasm gap

**DOI:** 10.1111/bjso.70141

**Published:** 2026-10-22

**Authors:** Tanja Oschatz, Verena Klein, Jennifer L. Piemonte, Roland Imhoff, Terri Conley

**Affiliations:** ^1^ Institute for Social and Legal Psychology Johannes Gutenberg University Mainz Germany; ^2^ School of Psychology University of Southampton Southampton UK; ^3^ Department of Psychology University of Illinois Urbana‐Champaign Illinois USA; ^4^ University of Michigan Ann Arbor Michigan USA

**Keywords:** gender inequality, orgasm, orgasm gap, sexual satisfaction, sexuality, system justification

## Abstract

The “orgasm gap”—the consistent disparity in orgasm rates in sexual encounters between women and men—represents a striking form of gender inequality. Though orgasms strongly predict women's sexual satisfaction, many women downplay their importance. To explain this paradox, we drew on System Justification Theory (SJT) to investigate whether and how legitimizing the orgasm gap sustains sexual inequality between women and men across four studies with U.S. participants (*N* = 1967). Study 1 introduced a novel measure of orgasm gap‐system justification (OGSJ), showing that stronger justification was associated with lower orgasm centrality and entitlement among women, and higher orgasm entitlement among men. Studies 1 and 2 provided evidence for SJT's palliative function: irregularly‐orgasmic women who endorsed OGSJ beliefs reported greater sexual satisfaction despite low orgasm frequency. Studies 2 and 3 (the latter experimental) demonstrated the motivated nature of OGSJ, as irregularly‐orgasmic women selectively endorsed system‐affirming explanations and judged system‐threatening information as less credible than system‐affirming information. In Study 4, stronger OGSJ was associated with reduced support for social change initiatives promoting orgasm equality among women and men. These findings highlight how system justification sustains sexual inequality, offering psychological comfort at the cost of structural change.

Akin to the pay gap observed between women and men, wherein women receive lower incomes than men on average, there is also an “orgasm gap,” wherein women experience fewer orgasms than men on average in mixed‐gender sexual encounters (Frederick et al., 2018). This gender disparity is both substantial and persistent: while men report reaching orgasm in 85%–95% of these sexual encounters, women report doing so only 40%–65% of the time—a gap that has been consistently documented in research over the past several decades (Gesselman et al., 2024; Kontula & Miettinen, 2016; McElroy & Perry, 2024). Contrary to common assumptions and lay beliefs (Klein & Conley, 2022; Wetzel & Sanchez, 2022), the orgasm gap is not rooted in innate differences between women and men. Physiologically, there are no gender differences in functional capacities to orgasm and both women and men reach orgasm in the same amount of time during masturbation (Gérard et al., 2020; Hite, 1979; Laan et al., 2021). Women's orgasm rates also change across contexts: they are higher with long‐term or familiar partners, in sexual encounters with other women, and in sexual encounters that include a variety of sexual behaviours (Belu, 2025; Dickman et al., 2025). This pattern underscores the role of social and relational factors in shaping gender disparities in orgasm frequency (for a review, see Mahar et al., 2020). In sum, the orgasm gap is not caused by anatomical or biological gender differences (Armstrong et al., 2012; Mahar et al., 2020). Nor does it exist merely because of lack of awareness of this inequity– women can clearly articulate the gap to researchers (Bell & McClelland, 2018; Nicolson & Burr, 2003). Neither is the orgasm inconsequential. Orgasms can make women's sexual encounters five to six times more enjoyable for them (Armstrong et al., 2012), but women often downplay the importance of orgasms to their satisfaction when asked directly (Mark et al., 2014; McClelland, 2011). This raises a crucial question: Is the claim that orgasms are less important to women a form of system justification? Perhaps it is a strategy women adopt to protect their sexual satisfaction by downplaying their own unmet needs, which are often perceived as less legitimate or natural because of gender norms that constrain female sexuality (Ouahid et al., 2025). Across four studies, we investigated how justifying the orgasm gap relates to women's sexual satisfaction and biased information processing, as well as to women's and men's feelings of orgasm entitlement and attitudes towards social change tackling sexual inequality.

## System justification and its palliative function

In the present research, we draw on System Justification Theory (SJT) and propose that the orgasm gap, just like the gender pay gap, reflects a hierarchy in which women are disadvantaged and men are advantaged. SJT provides a framework to understand the reasons why individuals endorse and perpetuate unequal social systems (Jost, 2019; Jost & Banaji, 1994). The theory posits that people are motivated to believe in the fairness and legitimacy of systems they depend on, even when these systems treat people unequally (Jost & Banaji, 1994; Vargas‐Salfate et al., 2018), thereby maintaining social hierarchies (Becker & Wright, 2011; Jost & van der Toorn, 2012). Specifically, gender‐specific system justification involves the belief that women and men have equal opportunities and therefore any status differences—like wage disparities—are natural, deserved, or fair (Jost & Kay, 2005). The present research extends and examines SJT in a novel context—the orgasm gap.

What might motivate advantaged and disadvantaged group members to rationalize the gender orgasm gap? Among men—the advantaged group—rationalizing the orgasm gap serves a straightforward palliative function. By reframing unequal outcomes as legitimate or appropriate, system‐justifying beliefs can reduce feelings of guilt or defensiveness associated with benefiting from the status quo, as well as reduce perceived need for intervention to improve the situation (Jost et al., 2008; Jost & Hunyady, 2003). In the context of the orgasm gap, these beliefs may manifest through increased male entitlement to orgasms—that is, perceptions that men are inherently more deserving of orgasms compared to women and that men's sexual pleasure should take priority during sexual behaviour (Klein & Conley, 2022; Willis et al., 2018). These beliefs may also buffer threats to masculinity (Chadwick & van Anders, 2017) by externalizing or normalizing unequal orgasm outcomes, thereby reducing the implication that women's lack of orgasm reflects men's sexual inadequacy (Mahar et al., 2020).

Meanwhile, the reasons for members of disadvantaged groups to hold system‐justifying beliefs are complex, given that they must accept their inequity to perceive the system as legitimate. Although women may find sexual encounters to be pleasurable even if they do not orgasm (Laumann et al., 1994; Salisbury & Fisher, 2014), several studies have identified orgasm as the most significant predictor of women's sexual satisfaction (Armstrong et al., 2012; Dienberg et al., 2023; Frederick et al., 2018; Janssen et al., 2025; Leonhardt et al., 2018; Wetzel et al., 2022). Yet, women regularly minimize their orgasm importance to researchers (see page 3 of Piemonte et al., 2019 for an overview) and to themselves (Klein & Conley, 2022).

Particularly in qualitative studies, women express that their pleasure is broader than orgasm, encompassing emotional or affectionate aspects of sex (Bell & McClelland, 2018; Gavey et al., 1999). For example, McClelland (2011) found that none of her female interview participants (*n* = 41) explicitly invoked orgasm as the primary criterion for their sexual satisfaction, instead opting for relational aspects of a sexual encounter—such as feeling close to their partner. In a separate study on sexual pleasure, one interviewee specifies, “I am physical I like to hug and hold hands and have that comforting relationship. I wouldn't say that orgasms come anywhere near the top of the list. That is an extra…” (Nicolson & Burr, 2003, p. 1740). Quantitative studies corroborate these claims: women place relatively less value on orgasm as compared to other aspects of sexual satisfaction, and in comparison to men (Mark et al., 2014; Muehlenhard & Shippee, 2010).

System Justification Theory (SJT) suggests that disadvantaged group members engage in this seemingly paradoxical method of thinking as a protective mechanism, or because it serves a palliative function. Individuals align their beliefs with unequal outcomes to reduce feelings of discomfort, uncertainty, or injustice that could arise if they acknowledge the unfair system (Day et al., 2011; Jost & Banaji, 1994). This belief adjustment helps individuals meet epistemic needs for certainty and coherence, and/or relational needs for a shared reality, which in turn can enhance short‐term well‐being (Hardin & Higgins, 1996; Jost et al., 2008; Jost & Hunyady, 2005; Vargas‐Salfate et al., 2018). Regarding the orgasm gap, women may downplay the importance of orgasms to mitigate distress associated with not experiencing them as frequently as their partners or as they desire, thereby maintaining a sense of sexual satisfaction (Laan et al., 2021). Such reactions to inequality are not unique to sexual contexts: women who earn less than men often shift their focus from income to job satisfaction (Bylsma & Major, 1994), and new mothers frequently revise their career goals in line with traditional roles (Schober & Scott, 2012). A cross‐cultural study involving participants from 23 countries found that women who denied the existence of gender‐based discrimination reported higher subjective well‐being (Napier et al., 2020). Across domains, minimizing gender inequality appears to serve palliative functions for women (Harding & Sibley, 2013; Suppes et al., 2019).

More broadly, when such palliative processes operate across both disadvantaged and advantaged group members, the individual relief they provide is accompanied by a collective cost: obstructing progress towards rectifying the unfair system. Legitimizing unequal outcomes in everyday sexual experiences inadvertently reinforces systemic gender inequality, stabilizes the restrictive status quo, and hinders potential for collective action in support of social change (Jost et al., 2010; Osborne et al., 2018).

## Women's justification strategies

As we have reviewed, justifying inequalities is a motivational process rather than a passive one, and individuals engage in various cognitive strategies to justify the social systems in which they live (Jost et al., 2010). One such strategy involves adjusting one's expectations to align with unequal outcomes (de Jong et al., 2018; Farvid, 2014; Wetzel et al., 2022). Recent research has captured evidence that women may in fact alter their expectations for sex with men to omit their orgasms (Wetzel et al., 2022; Wetzel et al., 2026), and to more generally prepare for reduced sexual pleasure (Conley et al., 2014; Conley & Klein, 2022). Furthermore, women report lower orgasm centrality (the extent to which orgasm is central to a person's sexual experience; Rubin et al., 2019), and less orgasm entitlement (the belief that one deserves to orgasm during sex) compared to their male partners (Klein & Conley, 2022). Rather than indicating a reduced desire for orgasm, these patterns may reflect an adaptive adjustment to sexual expectations that vary by gender. That is, perhaps women recalibrate their standards for pleasure to align with the gendered boundaries of heteronormative sexual dynamics.

Another motivational strategy involves biased information processing, or the tendency to selectively attend to and endorse information that supports an existing system while discounting evidence that challenges it (Hennes et al., 2016; Jost et al., 2010; Kunda, 1990; Ledgerwood et al., 2011). Selective information processing can contribute to false consciousness—the tendency to hold beliefs that undermine one's own interests or those of one's group (thus perpetuating inequality; Jost & Banaji, 1994). In the case of the orgasm gap, several false beliefs about gender and sexuality likely sustain this inequality. For instance, it is common for people to report limited knowledge about female sexual anatomy and orgasmic capacity (Dienberg et al., 2023; Séguin & Blais, 2019), with researchers documenting the widely held misconception that women's orgasms are innately elusive and anatomically more “complicated” as compared to men's (Conley & Klein, 2022; Mahar et al., 2020; Mulroy et al., 2025; Séguin & Blais, 2021; Wetzel & Sanchez, 2024). As such, individuals may genuinely perceive the orgasm gap as biologically determined, thus facilitating system‐justifying interpretations of the status quo. Indeed, one study found that young adults were more likely to attribute the orgasm gap to “biology” rather than acknowledging the significant influence of sociocultural factors (Wetzel & Sanchez, 2022). It is possible that with the predominance of essentialist narratives like these, women pursue their epistemic and relational needs by over‐defining their sexual well‐being in less embodied ways, such as through relational intimacy or emotional closeness (Chadwick, 2024; Laan et al., 2021).

### The present study

Applying SJT to small‐scale systems like intimate and sexual relationships is a meaningful extension of prior research, which has largely focused on macro‐level systems such as political institutions or economic structures (Jost, 2019). The current research takes a social psychological approach to better understand how women (and men) may come to rationalize or legitimize the orgasm gap. We understand the orgasm gap as a manifestation of broader gendered systems of inequality that are enacted and stabilized within intimate, often dyadic contexts. Consistent with this perspective, SJT defines a “system” as any organized and closely‐intertwined network of social relations, encompassing both macro‐level structures (e.g. society) and micro‐level contexts such as families or romantic dyads (Friesen et al., 2019; Jost et al., 2015; Wakslak et al., 2011). To date, only two studies have examined system justification within romantic relationships, showing that men defend traditional relationship norms when their status is threatened and that women justify gender inequality more when their relationships are perceived positively (Day et al., 2011; Sobol‐Sarag et al., 2023). Building on this work, we extend the application of SJT to the system of sexual relationships. We suggest that in mixed‐gender contexts, both women and men are motivated to justify the orgasm gap and perceive the prevailing system as fair, natural, and legitimate. We further suggest that these beliefs facilitate the maintenance of sufficient sexual satisfaction and ultimately undermine support for social changes in service of gender equality.

In four studies involving U.S. participants (total *N* = 1967) we tested predictions derived from SJT in the context of the orgasm gap. Study 1 (pre‐registered) introduced a novel measure of orgasm gap‐system justification (OGSJ) and examined its relationship to motivational strategies thought to sustain the orgasm gap (here, feelings of orgasm entitlement and orgasm centrality). We also explored the palliative function of system justification by examining whether higher endorsement of OGSJ beliefs predicted greater sexual satisfaction, particularly among the most disadvantaged group: irregularly‐orgasmic women (that is, women who do not usually orgasm during partnered sex with men). In Study 2, we aimed to replicate the palliative component of Study 1, using an alternative measure of orgasm frequency (orgasm consistency). We further investigated additional motivational strategies women may use to justify the orgasm gap by examining patterns of biased information processing. Specifically, we tested whether irregularly‐orgasmic women, when given a choice, prefer narratives that support rather than challenge the legitimacy of the orgasm gap.

In Study 3, we extended this line of inquiry by using an experimental design to examine the effects of exposure to system‐affirming versus system‐threatening narratives on women's perceptions of gender inequality. We assessed which type of narrative is seen as more plausible among women with infrequent orgasms. Finally, in Study 4, we assessed the effects of OGSJ on attitudes towards social change. We tested whether endorsement of justification beliefs predicted lower support for interventions aimed at reducing the orgasm gap, both among women and men.

#### Transparency statement

All data are available at the OSF and can be accessed at: https://osf.io/4afb6/overview. Data were analysed using SPSS, version 27. Study 1's design and analytical plan were pre‐registered. Studies 2, 3, and 4 were not. All decisions related to analyses were made prior to examining the data. We report how we determined our sample size, all data exclusions, all manipulations, and all measures in the study. The study protocols were reviewed and deemed exempt from oversight by the Ethics Committee of the University of Michigan.

## STUDY 1

We had three objectives for Study 1. First, we sought to establish the reliability and construct validity of the newly adapted OGSJ scale. Based on prior SJT research, we predicted that OGSJ would positively correlate with political conservatism and general gender‐specific system justification (Jost, 2019).

Second, we examined one motivational strategy of SJT: adjusted expectations. If women's relatively lower orgasm centrality and entitlement (as compared to men) serve to help them expect and accept sexual relationships with unequal orgasm rates, these variables should also be associated with OGSJ (Klein & Conley, 2022; Rubin et al., 2019). We hypothesized that higher OGSJ would be associated with lower orgasm centrality and entitlement among women, and with greater orgasm entitlement among men. As men orgasm during most heterosexual encounters (Frederick et al., 2018), we expected limited variability in orgasm centrality among men and, therefore, little predictive value of OGSJ for this outcome.

Third, we tested whether OGSJ serves a palliative function for women. Specifically, we hypothesized that OGSJ would buffer against drops in sexual satisfaction among irregularly‐orgasmic women. In contrast, we predicted that the buffering effect would diminish for regularly‐orgasmic women. We therefore tested a model with orgasm frequency predicting sexual satisfaction and OGSJ as a moderator. This study was pre‐registered: https://osf.io/4u2d8.

## METHOD

### Participants

All power analyses throughout the paper were performed using G*Power (Faul et al., 2009). An a priori power analysis was conducted for the planned moderation model using women participants, assuming a small interaction effect (*f* ^2^ = .03). The analysis indicated that a sample size of approximately 265 women would be required to achieve 80% power at α = .05.^1^ To obtain comparable precision for men and to allow for an anticipated 10% exclusion/attrition rate, we therefore targeted 295 per gender (planned total *N* = 590).

We recruited 629 U.S. adults via Amazon Mechanical Turk in 2021.^2^ Based on our pre‐registered exclusion criteria—applied consistently across studies—we excluded participants who did not identify as cisgender or who identified as having exclusively same‐sex attractions, as well as participants with incomplete data sets. We included both heterosexual and bisexual participants, as our focus was on individuals for whom the orgasm gap in mixed‐gender contexts is relevant, and prior research indicates that orgasm frequencies among bisexual and heterosexual women are comparable (Frederick et al., 2018). We excluded 28 participants due to incomplete data, three who did not identify as either women or men, and 43 who did not identify as hetero‐ or bisexual, resulting in a final sample of 555 participants (44% women). Participant ages ranged from 18 to 77 (*M* = 40.63, SD = 11. 38). Each participant was compensated 80 cents for the 5‐min study. Please see Table 1 for detailed sample descriptions for all studies.

**TABLE 1:** Sociodemographic characteristics of participants across all studies.

	Study 1		Study 2		Study 3		Study 4	
	*N*	%	*N*	%	*N*	%	*N*	%
Gender								
Women	246	44.3	245	100	329	100	415	49.6
Men	309	55.7	0	0	0	0	421	50.4
Sexual orientation								
Heterosexual	535	96.4	212	86.5	296	90.0	778	93.1
Bisexual	20	3.6	33	13.5	33	10.0	58	6.9
Ethnicity								
African American//Black	40	7.2	26	10.6	30	9.1	79	9.4
Asian American/Asian	60	10.8	17	6.9	21	6.4	95	11.4
European American/White	413	74.4	188	76.7	257	78.1	558	66.7
Hispanic/Latina	27	4.9	5	2.0	11	3.3	59	7.1
Middle Eastern	4	0.7	1	0.4	0	0	7	0.8
Native/American Indian	2	0.4	2	0.8	3	0.9	5	0.6
Multi‐ethnoracial	9	1.6	6	2.4	7	2.1	33	3.9
Education								
Less than high school	3	0.5	3	1.2	3	0.9	5	0.6
High school diploma	103	18.6	63	25.7	81	24.6	230	27.5
2‐year college/university degree	71	12.8	48	19.6	66	20.1	105	12.6
4‐year college/university degree	260	46.8	103	42.0	133	40.4	354	42.1
Master's degree	99	17.8	19	7.8	38	11.6	114	13.6
Professional degree (JD, MD)	9	1.6	8	3.3	5	1.5	17	2.0
Doctoral degree	10	1.8	1	0.4	3	0.9	13	1.6
Relationship status								
Single	158	28.5	68	27.8	86	26.1	272	32.5
In Relationship	397	71.5	72.2	72.2	243	73.9	564	67.5

### Procedure

Participants were instructed to respond to a series of questions and scales regarding their sexual experiences, orgasm attitudes, and gender beliefs. The different measures were displayed in random sequences across participants to control for order effects.

### Measures

#### Orgasm gap‐system justification

We adapted the Acceptance of Gender Income Inequality Scale (Connor & Fiske, 2019) to measure system justifying beliefs about the orgasm gap, as no existing validated measure captures this construct. The original scale was designed to measure the justification of gender‐based income inequality. We selected this scale because it was explicitly developed to assess the rationalization of a specific gender inequality (i.e., gender pay gap) and opposition to greater equality (i.e. pay equality) as expressions of system justification. Moreover, unlike broader gender system justification measures (e.g. Jost & Kay, 2005), which assess broad beliefs about the fairness of gender relations (e.g., family roles, societal opportunities), it focuses on a specific manifestation of gender inequality, making it particularly well suited for adaptation to the orgasm gap while requiring only minimal wording changes. We retained the original item structure and wording, changing only the type of inequality. Items referring to income inequality between women and men were adapted to focus on differences in orgasm frequency. For example, the item “Differences in income between men and women are too large” was adapted to “Differences in orgasm frequency between men and women are too large.”

Participants read the following introduction: “Research shows that men have more orgasms than women – a phenomenon known as the orgasm gap. One study found that for every orgasm a woman has, her male partner has about three (Wade, 2015).” Participants were then asked to indicate their agreement on seven items (e.g., “Differences in orgasm frequency between men and women are justified.”) using a six‐point Likert scale (1 = strongly disagree, 6 = strongly agree).

A confirmatory factor analysis indicated that one item showed a comparatively low factor loading and was therefore excluded. The resulting 6‐item scale demonstrated acceptable model fit (CFI = .933, SRMR = .076) and internal consistency (α = .85).

Model fit indices for both the initial 7‐item model and the final 6‐item model, as well as standardized factor loadings of all items, are reported in the Supporting Information on OSF. For the full scale please see the study protocol on the OSF: https://osf.io/4afb6/files/37dbs.

#### Gender‐specific system justification

The Gender‐Specific System Justification Scale assessed participants' rationalization of the gender dynamics that exist in society (Jost & Kay, 2005; e.g., “In general, relations between men and women are fair”). The measure includes eight items that are rated on a nine‐point Likert scale (1 = strongly disagree, 9 = strongly agree, α = .89).

#### Political orientation

We assessed political orientation with the standard libcon item from the U.S. National Election Studies: “When it comes to politics, do you usually think of yourself as…?” Responses ranged from 1 = extremely liberal to 7 = extremely conservative.

#### Orgasm frequency

Orgasm frequency was measured with the following item: “Generally speaking, please indicate how often you orgasm during your sexual encounters with a partner.” Participants indicated their answer on a five‐point Likert scale from 1 = never to 5 = always.

#### Orgasm centrality

Participants indicated the centrality of orgasming during sexual encounters with a partner (Rubin et al., 2019). Participants were asked, “Think back on all the times you engaged in sexual activity with a partner…” and were then prompted to indicate their agreement with four items (e.g.: “I was interested in orgasming”). Answers were indicated on a six‐point Likert scale (1 = strongly disagree, 6 = strongly agree, α = .89).

#### Orgasm entitlement

We adapted four items to measure whether participants valued their own or their partner's orgasm more (Klein & Conley, 2022; Klein et al., 2024; e.g., “Generally speaking, during a sexual encounter whose orgasm is more important?”). Answers were indicated on a Likert scale ranging from 1 = my partner's orgasm to 8 = my orgasm. Hence higher numbers represented stronger feelings of orgasm entitlement (α = .89).

#### Sexual satisfaction

We measured sexual satisfaction with the Global Measure of Sexual Satisfaction of the Interpersonal Exchange Model of Sexual Satisfaction Questionnaire (Lawrance & Byers, 1995). Participants rated their sex life on a seven‐point Likert scale on five different dimensions: *good‐bad*, *pleasant‐unpleasant*, *positive–negative*, *satisfying‐unsatisfying*, and *valuable‐worthless* (α = .89).

## RESULTS AND DISCUSSION

We first verified that the focal inequality was present in our data. As expected, the orgasm gap was evident in our sample: men reported significantly higher orgasm frequency (*M* = 4.34, SD = 0.73) compared to women (*M* = 3.40, SD = 1.06), *t*(553) = −11.81, *p* < .001, *d* = 1.05.

### Aim 1: Validating the OGSJ scale

The OGSJ scale was highly correlated with both gender‐specific system justification (*r* = .496, *p* < .001) and with a conservative political orientation (*r* = .309, *p* < .001), supporting its validity as a status quo‐supporting construct. All correlations for measures of this study can be found in Table 2.

**TABLE 2:** Variable correlations by participant gender.

	1	2	3	4	5	6
(1) O frequency	—	.477**	.191*	.158	−.015	.248**
(2) O centrality	.568**	—	.283**	.122	−.061	.126*
(3) O entitlement	.309**	.484**	—	.113	.312**	−.018
(4) Gender SJ^a^	.128	.395**	−.113	—	.419**	.257**
(5) OGSJ	.071	−.177**	−.275**	.568**	—	−.072
(6) Sexual satis.	.562**	.244**	.036	.012	.084	—
Women *M* (SD)	3.40 (1.06)	4.39 (1.24)	3.65 (1.47)	4.97 (1.77)	3.17 (1.07)	5.24 (1.46)
Men *M* (SD)	4.34 (0.73)	5.17 (0.88)	3.76 (1.48)	5.35 (1.77)	3.23 (1.04)	5.30 (1.45)

*Note*: Women (*n* = 246) are below the diagonal, men (*n* = 309) are above.

^a^ Due to a coding error, the gender‐specific system justification measure was only administered to a subset of the sample (*N* = 219).

** *p* < .01.

* *p* < .05.

### Aim 2: OGSJ And its association with orgasm centrality and entitlement by gender

#### Women

Among women, OGSJ was negatively associated with orgasm centrality (*r* = −.18, *p* < .01) and orgasm entitlement (*r* = −.28, *p* < .01), indicating that the more women endorse OGSJ, the less central orgasm is to their sexual satisfaction and the less entitled to orgasm they feel.

#### Men

For men, OGSJ was positively related to orgasm entitlement (*r* = .31, *p* < .01), indicating that the more men justified the orgasm gap, the more they preferred their own orgasm compared to their partner's (see Table 2).

### Aim 3: Palliative effect of OGSJ in women

To test the relationship between orgasm frequency, OGSJ, and their interaction on women's sexual satisfaction, we conducted a moderation analysis using the PROCESS‐macro (Hayes, 2018). The overall model was significant, *F*(2, 244) = 44.19, *p* < .001, accounting for approximately 35% of the variance in sexual satisfaction. Importantly, the interaction term reached significance, *B* = −0.22, SE = 0.06, *t*(244) = −3.67, *p* < .001, 95% CI [−0.34, −0.10]. This illustrates the moderating effect of OGSJ on the relationship between orgasm frequency and sexual satisfaction, such that it was stronger for participants who reported low OGSJ (1 SD below the mean), *B* = 1.03, SE = 0.10, *t*(244) = 10.22, *p* < .001, 95% CI [0.83, 1.23], than for those who reported high OGSJ (1 SD above the mean), *B* = 0.51, SE = 0.10, *t*(244) = 5.12, *p* ≤ .001, 95% CI [0.31, 0.70].

The interaction term's effect size was *f* ^2^ = 0.06 (see Figure 1). The main effects of orgasm frequency on sexual satisfaction, *B* = 1.47, SE = 0.20, *t*(244) = 7.18, *p* < .001, 95% CI [1.07, 1.88], and for OGSJ, *B* = 0.82, SE = 0.22, *t*(244) = 3.76, *p* < .001, 95% CI [0.39, 1.24], should therefore be interpreted in light of this interaction.

These results showed initial evidence of the palliative effect of system justification beliefs on sexual satisfaction in women. For women low in OGSJ, more frequent orgasms were strongly linked to higher sexual satisfaction. In contrast, for women high in OGSJ, this link was much weaker. This suggests that among irregularly‐orgasmic women, those who endorse OGSJ beliefs felt more satisfied with their sex lives than those who do not hold OGSJ beliefs—consistent with a palliative effect of OGSJ (see Figure 1).

**FIGURE 1:**
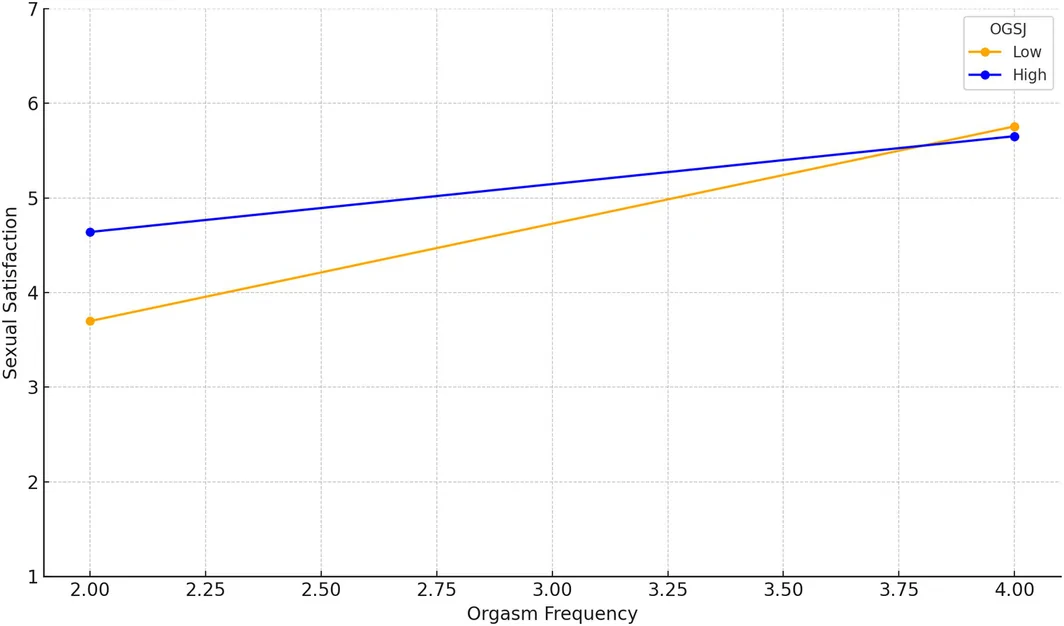
Orgasm gap‐system justification moderates the relationship between orgasm frequency and sexual satisfaction.

## STUDY 2

Study 2 aimed to replicate and extend the findings of Study 1. First, we sought to replicate the palliative function of system justification using a simplified, dichotomous measure of orgasm consistency differentiating between women who usually orgasm during partnered sex and women who usually do not. Second, we wanted to test another core tenet of SJT: that justifying beliefs are motivated and actively maintained. To this end, Study 2 examined whether women engage in biased information processing, specifically whether they are more likely to choose system‐affirming narratives (e.g., narratives that provide rationale for unequal orgasm rates between women and men) over system‐threatening narratives (e.g., positions that counter the orgasm gap), and whether women's own personal orgasm frequencies moderate this preference.

Our approach draws on literature showing that system justification can promote endorsement of essentialist explanations for gender differences, because such explanations make the social hierarchy appear natural and immutable. When gender differences are seen as inherent, unequal outcomes (e.g., pay gaps) are less likely to be viewed as injustice and more as the result of fixed, natural differences. In this way, essentialist beliefs help legitimize and maintain existing gender patterns, consistent with system‐justifying motives (Brescoll et al., 2013; Morton et al., 2009; Skewes et al., 2018). Supporting this, Kray et al. (2017) demonstrated that individuals who endorse entity theories of gender (i.e., viewing gender roles as fixed and biologically determined), also reported higher levels of gender system justification, indicating greater support for the status quo in gender relations. Accordingly, we predicted that irregularly‐orgasmic women would be more likely to endorse explanations that naturalize or downplay the orgasm gap (i.e., biological essentialism), thereby justifying the existing inequality.

## METHOD

### Participants

The current study focused exclusively on women. An a priori power analysis was conducted for the planned moderation model, using the interaction effect size observed in Study 1 (*f* ^2^ = 0.05). Based on this effect size, a power analysis indicated that a minimum sample of approximately 159 participants would be required to achieve 80% power at an alpha level of 0.05. Due to a high fall‐out rate observed in Study 1, we aimed for at least 200 participants. In addition, a somewhat larger sample was collected to ensure sufficient group sizes of regularly and irregularly‐orgasmic women. A total of 259 participants were recruited on Mechanical Turk in 2021, exceeding the threshold of the power analysis. We excluded 14 participants as they did not fulfil the inclusion criteria (four were not female, 10 were not hetero‐ or bisexual), resulting in a final sample of 245 women (*M*_age_ = 42.18, SD_age_ = 11.89, range = 22–74). Participants were compensated 50 cents for the 4‐min study.

### Procedure

Participants were presented with two pairs of research findings and asked to select the one they found more credible in each case. They then completed a brief survey measuring OGSJ, additional sexual outcomes, and demographic information.

### Materials

Participants were instructed to pick between two research findings in two separate pairings. Each pairing focused on a distinct narrative related to women's orgasms. Participants were asked, “Which of the two research findings on orgasm do you find more plausible?”

For the first pairing, participants could choose between the following two options: (1) Orgasming reliably is the most important indicator for a woman's sexual satisfaction, or (2) Feeling close to her partner is the most important indicator of a woman's sexual satisfaction.

The system‐affirming option (2) reflects a longstanding cultural belief that women are more sexually satisfied by emotional fulfilment or relational closeness than by physical fulfilment (DeLamater, 1987; Séguin & Blais, 2019). Despite robust evidence that orgasm is the superior predictor of women's sexual satisfaction (Dienberg et al., 2023; Janssen et al., 2025; Klapilová et al., 2015; Leonhardt et al., 2018; Piemonte et al., 2019), this belief persists—reinforcing traditional gender stereotypes, ascribing women a nurturing, relational role and downplaying their entitlement to physical pleasure (Klein & Conley, 2022; Oschatz et al., 2025).

The second pairing offered the following two options: (1) Men and women differ in their biological capacity for orgasms, or (2) Men and women do not differ in their biological capacity for orgasms.

Here, the system‐affirming option (1) reflects biological essentialism—the idea that gendered differences in orgasm are rooted in immutable biology. This aligns with a broader pattern in system justification, where inequality is explained through supposed natural differences, thus reducing perceived injustice (Brescoll et al., 2013).

### Measures

#### Orgasm gap‐system justification

We measured OGSJ as in Study 1 (Cronbach's α = 0.90).

#### Orgasm consistency

To enable clearer group comparisons, in this study we used a dichotomous measure of orgasm consistency to distinguish between women who regularly orgasm during partnered sex and those who do not. We measured orgasm consistency with the item “Generally speaking, I orgasm when I have sex with a partner.” (1 = yes, 2 = no). Participants who responded “no” are referred to as irregularly‐orgasmic women, while those who responded “yes” are referred to as regularly‐orgasmic throughout the study. Of the total sample, 35.9% (*n* = 88) of women belonged to the irregularly‐orgasmic group, while 64.1% (*n* = 157) belonged to the regularly‐orgasmic group.

#### Sexual satisfaction

Sexual satisfaction was measured using the same items as in Study 1, though responses in this study were recorded on a 5‐point scale rather than a 7‐point scale. Cronbach's α for this sample was 0.97.

## RESULTS AND DISCUSSION

### Aim 1: Replicating the palliative effect

To replicate the results of Study 1 we again conducted a moderation analysis with the PROCESS‐macro (Hayes, 2018). The overall model was significant, *F*(3, 241) = 15.07, *p* < .001, accounting for approximately 15.8% of the variance in sexual satisfaction. The interaction term reached marginal significance, *B* = 0.25, SE = 0.13, *t*(241) = 1.88, *p* = .062, 95% CI [−0.01, 0.50], *f* ^2^ = 0.02. Consistent with Study 1, when OGSJ was low (1 SD below the mean), the relationship between orgasm frequency and sexual satisfaction was stronger. Meanwhile, when OGSJ was high, orgasm frequency was a weaker predictor of sexual satisfaction. Sexual satisfaction scores were significantly different between regularly‐ and irregularly‐orgasmic women, *B* = −1.26, SE = 0.22, *t*(241) = −5.70, *p* < .001, 95% CI [−1.69, −0.82]. When OGSJ was high (1 SD above the mean), the differences remained significant but with a smaller effect, *B* = −0.73, SE = 0.19, *t*(241) = −3.78, *p* < .001, 95% CI [−1.10, −0.35].

The main effect of orgasm consistency on sexual satisfaction was significant, *B* = −1.75, SE = 0.62, *t*(241) = −3.88, *p* < .001, 95% CI [−2.64, −0.86], while the main effect for OGSJ was not significant (*p* = .181). As in Study 1, we observed a pattern consistent with the palliative effect of OGSJ (see Figure 2). Although the interaction between orgasm consistency and OGSJ did not reach conventional levels of statistical significance (*p* = .062), the estimated effect was similar in direction and magnitude to that observed in the preregistered Study 1 (see Figure 2). Thus, the results are consistent with, but do not provide independent statistical support for, the proposed palliative effect of OGSJ. One possible reason for this attenuation is the dichotomous operationalization of orgasm consistency, which likely reduced variance compared to the continuous measure used in Study 1. Future research should use more fine‐grained measures of orgasm consistency to better capture variability and estimate this interaction more precisely.

**FIGURE 2:**
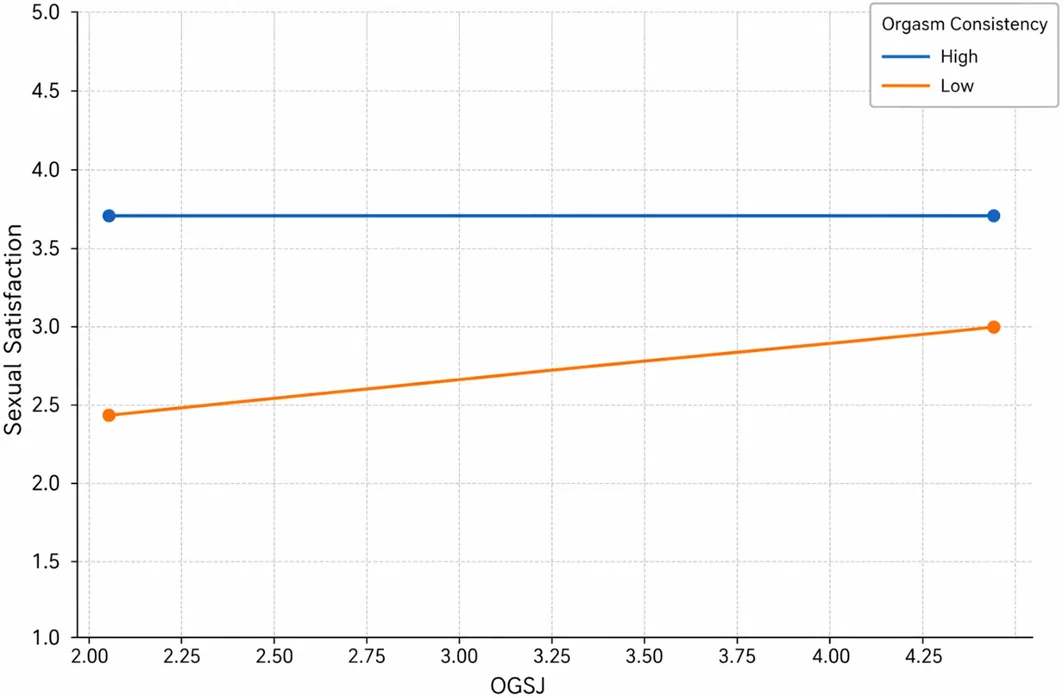
Effect of OGSJ on the relationship between orgasm consistency and sexual satisfaction.

### Aim 2: Narrative preference and orgasm consistency

Before testing Study 2's main hypotheses, we conducted preliminary analyses to evaluate the validity of the stimuli. Specifically, we examined whether prioritizing emotional closeness and endorsing biological differences in orgasm capacity were associated with OGSJ using two independent‐samples *t*‐tests. Women who prioritized partner closeness as the most important indicator of female sexual satisfaction reported significantly higher OGSJ beliefs (*M* = 3.32, SD = 1.14) than those who emphasized orgasm (*M* = 2.82, SD = 1.18), *t*(243) = −2.92, *p* = .004, *d* = 0.43. Similarly, women who endorsed biological differences in orgasm capacity reported higher OGSJ beliefs (*M* = 3.28, SD = 1.10) than those who rejected such differences (*M* = 2.82, SD = 1.29), *t*(62) = 2.17, *p* = .034, *d* = 0.40. Overall, higher OGSJ beliefs were linked to reduced emphasis on orgasm in sexual satisfaction and greater endorsement of biological explanations for orgasm differences.

To test our main hypotheses for Study 2, we conducted a chi‐square test of independence examining the first pair of legitimizing narratives. Regularly‐orgasmic women were more likely than irregularly‐orgasmic women to select the system‐threatening research (that orgasm is the most important indicator of women's sexual satisfaction), *χ* ^2^(1, *N* = 245) = 5.93, *p* = .015, *w* = 0.16. In contrast, irregularly‐orgasmic women were more likely than regularly‐orgasmic women to select the system‐affirming research that feeling close to their partner is the most relevant indicator. This is initial evidence that endorsing legitimizing myths about gendered sexuality corresponds to OGSJ among women with irregular orgasms. In contrast, regularly‐orgasmic women may be less susceptible to this form of justification.

However, there was no significant association between participants' orgasm consistency and the second pair of legitimizing narratives, *χ* ^2^(1, *N* = 245) = 2.02, *p* = .155, *w* = 0.09. Both groups of women preferred the research finding that women and men differ in their biological capacity for orgasms (as opposed to the research finding that women and men do not differ). Importantly, “regularly‐orgasmic” does not imply parity with male partners; even women who typically orgasm during partnered sex still often report doing so less often than their partners. This gap may help explain the continued appeal of some system‐justifying beliefs. Therefore, the absence of differences between irregularly and regularly‐orgasmic women in endorsement of biological explanations may reflect the pervasive cultural availability of essentialist accounts (Conley & Klein, 2022; Wetzel & Sanchez, 2024). Another possible explanation for this null finding is that participants may have interpreted “biological capacity” in the context of penetrative sex, as most people tend to equate “sex” with penetration (Fee et al., 2025; Peck et al., 2016). This idea itself may function as a system‐justifying narrative by normalizing sexual activities and even specific patterns of stimulation that facilitate the orgasm gap (because vaginal intercourse is reliably associated with orgasms for men, but not women).

## STUDY 3

Building on the correlational findings of Study 2, in Study 3 we aimed to capture experimental evidence for the motivated nature of orgasm gap‐system justification via biased information processing. We tested whether women would evaluate the credibility of research findings differently depending on whether the information affirms or threatens the legitimacy of the existing system. Participants read a research summary about orgasms and sexual satisfaction with a framing that either affirmed or threatened the system. We used the narratives from Study 2, so the stimuli in the system‐affirming condition described the orgasm gap as inevitable due to essential gender differences and suggested orgasms are peripheral to women's sexual satisfaction. Meanwhile, the system‐threatening condition challenged the legitimacy of the orgasm gap and highlighted the importance of orgasms for women's sexual satisfaction.

Given that system‐threatening information particularly motivates defence of the status quo when it is perceived as personally relevant (Jost, 2019), we expected an interaction between condition and participants' personal orgasm consistency. We predicted that irregularly‐orgasmic women in the system‐threatening condition would rate the research as less credible than regularly‐orgasmic women. We did not expect such differences in the system‐affirming condition. Additionally, we predicted that system‐affirming information would reinforce the psychological palliative function: irregularly‐orgasmic women should report higher sexual satisfaction after reading system‐affirming research compared to system‐threatening research. We did not have specific hypotheses about system justification among regularly‐orgasmic women given that in Study 2, they endorsed one legitimizing narrative and refuted the other^3^

## METHOD

### Participants

An a priori power analysis indicated that a sample of 328 participants would be sufficient to detect a small to medium effect size (*f* = 0.18) in a 2 × 2 between‐subjects ANOVA with 80% power at α = 0.05. We recruited 352 women via Mechanical Turk in 2021. We excluded 23 participants as they did not fulfil the inclusion criteria (three were not female, 20 were not hetero‐ or bisexual), resulting in a final sample of 329 women (*M*_age_ = 43.94, SD_age_ = 12.81, range = 21–71, US $1 compensation for an 8‐min survey).

### Procedure

The study involved a 2 (orgasm consistency: high vs. low) × 2 (system justification condition: system‐affirming vs. system‐threatening) between‐subjects design. After providing informed consent, participants first indicated whether they typically orgasm during sex with a partner. They were then randomly assigned to either the system‐affirming (*N* = 159) or system‐threatening condition (*N* = 170), where they were presented with one of two constructed journalistic excerpts presenting research findings. The distribution of orgasm consistency did not differ by condition, χ^2^(1, *N* = 329) = 0.75, *p* = .387. Following the manipulation, participants evaluated the research's credibility, completed a measure of sexual satisfaction, and responded to demographic questions.

#### Manipulation: Affirming vs. threatening the system

The summary in the system‐affirming condition read as follows:

Studies show that men and women are very different in their capacities of experiencing orgasm during sex. Thanks to anatomical differences, the female orgasm is much more tricky and complex compared to the male orgasm. Therefore, biologically speaking, it is much easier for men to reach orgasms than for women. Moreover, research shows that relationship aspects (e.g., feeling close to your partner) are the number one aspect of sexual satisfaction for women.

In the system‐threatening condition, the summary gave the following information:

Studies show that men and women are not different in their capacities of experiencing orgasm during sex. Women have the same anatomical and biological experiences with orgasm as men. Therefore, biologically speaking, there is no reason why men experience orgasm more frequently than women do. Moreover, research shows that orgasm is the number one aspect of sexual satisfaction for women.

### Measures

#### Orgasm consistency

Participants' general tendency to orgasm or not during sexual encounters was measured in the same way as in Study 2. Most women (72.6%, *n* = 239) were in the regularly‐orgasmic group while 27.4% (*n* = 90) were in the irregularly‐orgasmic group.^4^

#### Credibility index

After the manipulation, participants answered seven items to assess how credible they perceived the presented research results using the following adjectives: accurate, false (R), credible, biased (R), suspicious (R), simplistic (R), and trustworthy. Participants indicated their agreement on a 6‐point scale (1 = strongly disagree, 6 = strongly agree). These seven items were combined into a credibility index (Cronbach's α = 0.93).^5^

#### Sexual satisfaction

Sexual satisfaction was measured as in Study 2. Cronbach's α reached 0.97.

## RESULTS AND DISCUSSION

### Credibility index

We conducted a 2 × 2 ANOVA for each dependent variable. The predicted interaction between condition and orgasm consistency was significant (see Figure 3), *F*(1, 325) = 19.19, *p* < .001, η_p_^2^ = 0.055. In the system‐affirming condition, there was no significant difference in perceived credibility between irregularly‐orgasmic (*M* = 4.58, SD = 0.83) and regularly‐orgasmic women (*M* = 4.39, SD = 0.83), *t*(157) = −1.24, *p* = .108, *d* = −0.23, 95% CI [−0.49, 1.10]. In the system‐threatening condition, regularly‐orgasmic women judged the research to be significantly more credible (*M* = 3.84, SD = 1.13) compared to irregularly‐orgasmic women (*M* = 2.94, SD = 1.20), *t*(168) = 4.63, *p* < .001, *d* = 0.78, 95% CI [0.52, 1.28].

**FIGURE 3:**
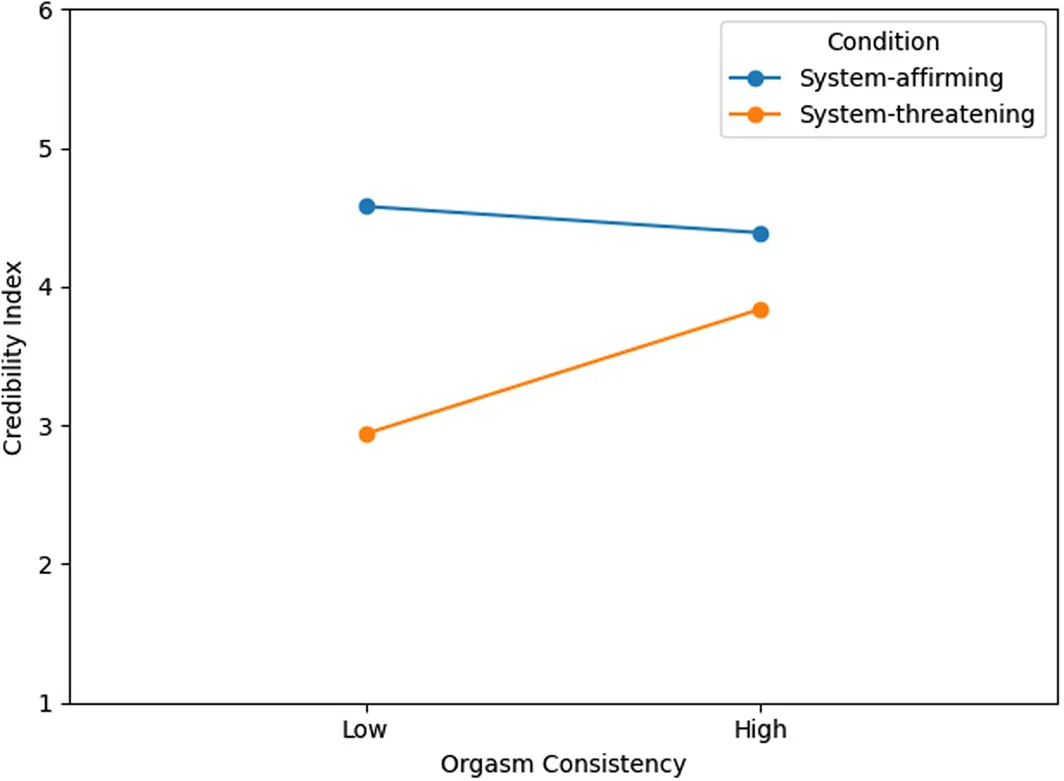
Interaction between condition and orgasm consistency on credibility judgement.

There was a main effect of condition on the credibility index, *F*(1, 325) = 75.50, *p* < .001, η_p_^2^ = 0.189, such that participants in the affirming condition (*M* = 4.44, SD = 0.83) reported higher credibility in the research than those in the threat condition (*M* = 3.58, SD = 1.22), *t*(327) = 7.40, *p* < .001, 95% CI [0.63, 1.09], *d* = 0.82. There was also a main effect of orgasm consistency on credibility index, *F*(1, 325) = 8.02, *p* = .005, η_p_^2^ = 0.024, such that regularly‐orgasmic women (*M* = 4.11, SD = 1.03) perceived a higher credibility of the research compared to irregularly‐orgasmic women (*M* = 3.67, SD = 1.33), *t*(327) = 3.22, *p* < .001, *d* = 0.82, 95% CI [0.17, 0.72]. However, these main effects should be interpreted with caution given the significant interaction.

This provides initial causal evidence that irregularly‐orgasmic women are motivated to justify the orgasm gap by delegitimizing system‐threatening information. Specifically, they rated research challenging the fairness and naturalness of the orgasm gap as significantly less credible than research affirming the orgasm gap. Meanwhile, no such effect was found for regularly‐orgasmic women.

### Sexual satisfaction

The interaction between condition and orgasm consistency also reached significance (see Figure 4), *F*(1, 325) = 6.88, *p* = .009, η_p_^2^ = 0.021. Irregularly‐orgasmic women reported more sexual satisfaction after reading system‐affirming information (*M* = 2.89, SD = 1.26) than after reading the system‐threatening information (*M* = 2.30, SD = 0.95), *t*(88) = 2.54, *p* = .006, *d* = 0.54, 95% CI [0.13, 1.06]. Regularly‐orgasmic women did not differ in their sexual satisfaction depending on whether they read system‐affirming information (*M* = 3.77, SD = 1.15) or the system‐threatening information (*M* = 3.90, SD = 1.08), *t*(237) = −0.90, *p* = .184, *d* = −0.12, 95% CI [−0.41, 0.15].

**FIGURE 4:**
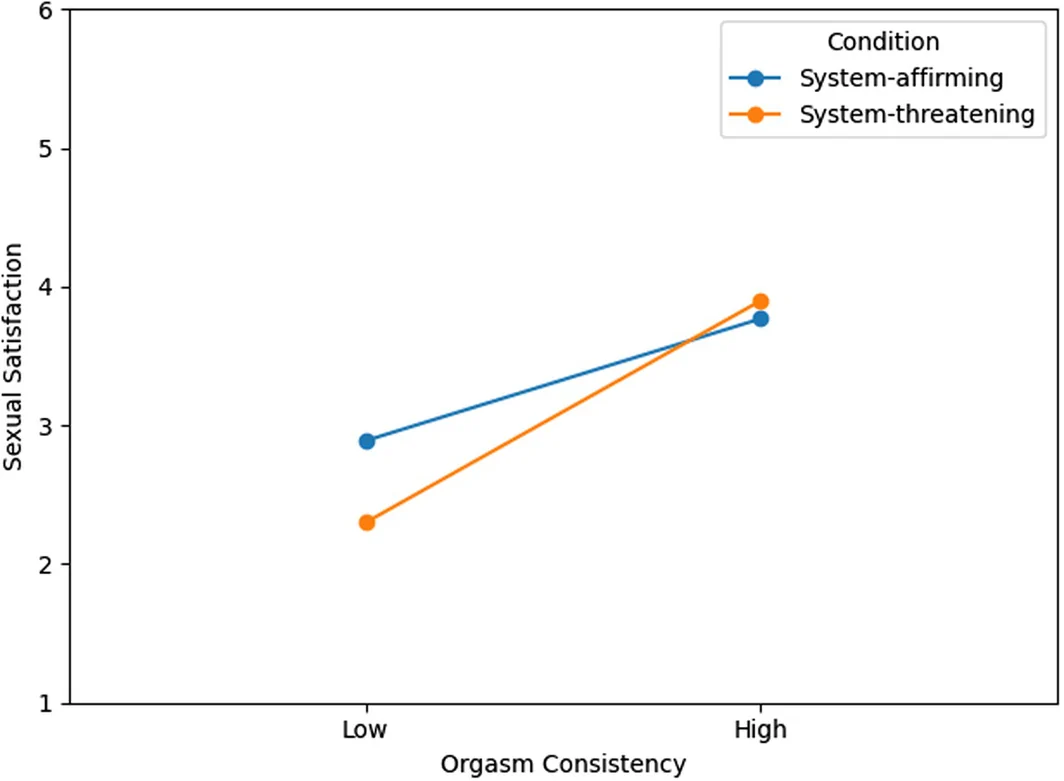
Interaction between condition and orgasm consistency on sexual satisfaction.

The results did not reveal a significant main effect of condition on sexual satisfaction, *F*(1, 325) = 2.82, *p* = .094, η_p_^2^ = 0.009. However, the main effect of orgasm consistency on sexual satisfaction was significant, *F*(1, 325) = 99.39, *p* < .001, η_p_^2^ = 0.198, indicating that satisfaction was higher among regularly‐orgasmic women (*M* = 3.83, SD = 1.11) than among irregularly‐orgasmic women (*M* = 2.56, SD = 1.13), *t*(327) = 9.18, *p* < .001, *d* = 1.14, 95% CI [1.00, 1.54].

These results show that exposure to system‐threatening information disrupted the palliative effect: irregularly‐orgasmic women reported lower sexual satisfaction after reading system‐challenging messages. This highlights the fragility of system justification as a coping strategy when the legitimacy of the status quo is questioned.^6^

## STUDY 4

One of the negative societal consequences of system justification is reduced motivation to engage in or support collective actions that challenge the unequal status quo and promote social change (Osborne et al., 2018). In Study 4, we therefore examined whether endorsing OGSJ beliefs is associated with lower motivation to support policy changes aimed at addressing gender inequality and closing the orgasm gap. We predicted that stronger OGSJ beliefs would predict reduced willingness—among both women and men—to engage in collective action or endorse policies promoting orgasm equality.

We also explored another possible strategy of SJT: denial of personal relevance. We asked how fair participants perceived the orgasm gap at both the dyadic level (within their own relationships) and the group or societal level.

## METHOD

### Participants

We recruited 879 participants via Mechanical Turk and Prolific in 2022 and 2023. We excluded 11 participants because they did not complete the variables of interest and an additional 30 participants because they did not fulfil the inclusion criteria (three were not female or male, 27 were not hetero‐ or bisexual), resulting in a final sample of 836 women and men (49.6% women). Participant age ranged from 18 to 93 years old (*M*_age_ = 38.61, SD_age_ = 13.30, with 70‐pence compensation for the 7‐min study).

### Procedure

Participants completed cross‐sectional online surveys with measures of OGSJ, policy support, fairness perceptions, and demographics.

### Materials

#### Orgasm gap‐system justification

We used the same measure of OGSJ as in the prior studies (Cronbach's α = 0.87).

#### Political orientation

We assessed political orientation in the same way as in Study 1.

#### Support for actions towards orgasm equality

We measured how much people would support actions being taken in society to make progress towards orgasm equality between women and men. We assessed this in two contexts: sex education policy and media advice. We intentionally wrote items suggesting specific material changes because people often endorse equality in principle, but not in practice (Brown et al., 2022). For example, individuals typically agree that they support equal opportunities for, and proportional representation of, underrepresented group members. But when posed with specific practices like using hiring quotas or investigating claims of discrimination, favourable attitudes drop (Crosby et al., 2006). We therefore included items describing relatively extreme measures taken in pursuit of orgasm equality.

Support for actions towards orgasm equality within sex education was measured with the following: *Current efforts have been observed to integrate sex‐positive sex education that stresses consent and pleasure in curricula for middle school. How much do you support this policy?* Support for actions towards orgasm equality within media advice was measured with the following: *A men's magazine recently suggested that during sex, guys should only climax if their partner does. How much do you support this advice?* Participants answered each item on a 6‐point scale ranging from 1 = don't support at all to 6 = support completely.

#### Support for gender equality

General support for gender equality was measured with three items (e.g. “I would sign an online petition to establish a law demanding minimum quotas for women in leading positions;” Becker & Wright, 2011). Participants indicated their intentions to participate in collective action on a 6‐point scale (1 = strongly disagree, 6 = strongly agree; Cronbach's α = 0.80).

##### Societal fairness perception

To measure how fair participants perceive the orgasm gap at the group level, we asked participants to rate their agreement with the following item: “The difference between how much men orgasm and how much women orgasm is fair.” The item was answered on a four‐point Likert scale (1 = strongly disagree, 4 = strongly agree).

##### Dyadic fairness perception

To assess participants' perceptions of orgasm distribution within their own relationships, they were asked to rate the following item: “The difference between how much I orgasm and how much my partner orgasms is fair.” This statement was rated on a four‐point Likert scale (1 = strongly disagree, 4 = strongly agree).

## RESULTS AND DISCUSSION

Please see Table 3 for all correlations for both genders.

**TABLE 3:** Correlations by participants' gender.

Variable	1	2	3	4	5	6
(1) OGSJ	—	−0.455**	−0.435**	−0.499**	0.572**	0.300**
(2) Sex education	−0.415**	—	0.274**	0.531**	−0.250**	−0.101*
(3) Media advice	−0.521**	0.210**	—	0.280**	−0.213**	−0.082
(4) Gender equality	−0.499**	0.452*	0.292**	—	−0.302**	−0.207**
(5) Fairness‐ Societal	0.633**	−0.218**	−0.309**	−0.307**	—	0.517**
(6) Fairness‐ Dyadic	0.308**	−0.053	−0.091	−0.060	0.343**	—
Women *M* (SD)	3.21 (1.15)	3.53 (1.88)	2.96 (1.68)	4.47 (1.56)	1.87 (0.89)	2.77 (1.12)
Men *M* (SD)	3.62 (1.02)	3.25 (1.82)	2.95 (1.66)	3.83 (1.62)	2.31 (0.92)	2.85 (0.87)

*Note*: Women (*n* = 417) are below the diagonal, men (*n* = 421) are above.

** *p* < .01.

* *p* < .05.

### Policy support

We conducted multiple regressions separately for women and men to examine the effects of OGSJ on support for actions towards orgasm equality (see Table 4 for all results). Because OGSJ was highly negatively correlated with support for general gender equality, we included the latter as an additional variable in our regression model to rule out the possibility that the lack of support for orgasm equality policies is explained by a general lack of support for gender equality. We also included political orientation given that conservatism is linked to less support for gender equality (Sevincer et al., 2023).

**TABLE 4:** Multiple regression results predicting support for orgasm equality in sex education and media advice among women and men.

Sample	Outcome	Predictor	*ß*	SE	*t*	*p*	Model summary
Women	Sex Education						*F*(3, 411) = 69.73, *p* < .001, *R* ^2^ = .34, *f* ^2^ = 0.52
		OGSJ	−0.16	0.08	−3.42	<.001	
		Support for Gender Equality	0.17	0.06	3.40	<.001	
		Political Orientation	−0.36	0.05	−7.30	<.001	
Women	Media Advice						*F*(3, 411) = 52.69, *p* < .001, *R* ^2^ = .28, *f* ^2^ = 0.39
		OGSJ	−0.52	0.07	−10.41	<.001	
		Support for Gender Equality	0.08	0.06	1.63	.127	
		Political Orientation	0.09	0.05	1.72	.087	
Men	Sex Education						*F*(3, 417) = 75.80, *p* < .001, *R* ^2^ = .35, *f* ^2^ = 0.54
		OGSJ	−0.23	0.08	−5.01	<.001	
		Support for Gender Equality	0.34	0.05	7.01	<.001	
		Political Orientation	−0.17	0.05	−3.79	<.001	
Men	Media Advice						*F*(3, 417) = 35.77, *p* < .001, *R* ^2^ = .21, *f* ^2^ = 0.27
		OGSJ	−0.41	0.08	−8.05	<.001	
		Support for Gender Equality	0.13	0.06	2.36	.019	
		Political Orientation	0.11	0.05	2.32	.021	

For both women and men, OGSJ was a negative predictor of support for sex‐positive sex education alongside the other two variables, while it was the sole negative predictor of support for media advice aimed at orgasm equality. These results indicate that OGSJ exerts a unique influence beyond broader ideological beliefs. Future research could test whether these effects vary among women by orgasm frequency, in line with the moderation patterns observed in Studies 1 and 2.

### Fairness perceptions and personal relevance

Although women generally perceived the distribution of orgasms within their own relationships as moderately fair (*M* = 2.77, SD = 1.12), a two‐sided *t*‐test showed that they rated the societal distribution of orgasms between women and men as significantly less fair (*M* = 1.87, SD = 0.89), *t*(414) = −15.83, *p* < .001, *d* = 0.78. The same pattern was observed for men: they rated the distribution of orgasms within their own relationships as significantly fairer (*M* = 2.85, SD = 0.87) than the societal distribution (*M* = 2.31, SD = 0.92), *t*(420) = −12.45, *p* < .001, *d* = 0.61.

The asymmetry in participants' fairness perceptions is consistent with classic research on the denial of personal disadvantage, which shows that individuals—even members of minority groups—acknowledge group‐level inequality while believing they themselves are unaffected (Crosby, 1984). This can be considered a subtle form of system justification (Napier et al., 2020). For example, when confronted with evidence of racial inequality, members of advantaged racial groups often deny their own privilege while still acknowledging systemic disparities (Taylor Phillips & Lowery, 2015). Likewise, women have been found to recognize gender discrimination at the institutional level while denying its personal relevance within domains such as education (Crosby et al., 1989; Foster et al., 2004). Among men, perceptions of fairness may also be reinforced by a sense of entitlement to positive outcomes, as prior work suggests that men tend to perceive themselves as more deserving of rewards (Pelham & Hetts, 2001).

## GENERAL DISCUSSION

Despite a growing body of literature on the orgasm gap (e.g., Gesselman et al., 2024; Mahar et al., 2020; McElroy & Perry, 2024), little research has explained why people—especially women—are so tolerant of this gender inequality. The present research examined SJT to explore how both women and men come to accept—and even legitimize—the persistent orgasm gap. This offers insight into an observed paradox: although women's sexual satisfaction is robustly linked to orgasm, they often minimize their orgasm value and entitlement (Klein & Conley, 2022; Wetzel et al., 2026). Prior sexuality research has speculated about why women who have sexual relationships with men often downplay the importance of having orgasms (e.g., Laan et al., 2021; Laan & Rellini, 2011). Our studies provide an integrated, theory‐driven explanation of this phenomenon. By extending SJT to the micro‐level, dyadic‐system of heterosexual relationships, we demonstrated that the seemingly private context of one's sexual relationship is connected to broader intergroup dynamics.

### Psychological comfort at a cost

Building on previous system justification research in domains such as racial or income inequality, we found that similar processes operated in the intimate domain of sexual relationships. That is, both women and men justified the orgasm gap in ways that sustained sexual inequality. Many studies have found that members of disadvantaged groups report increased well‐being or satisfaction when they endorse system justification beliefs, representing the palliative function of system justification (Harding & Sibley, 2013; Napier et al., 2020; Suppes et al., 2019). We demonstrated the same statistical relationship, especially among women who report lower orgasm rates during partnered sex. Among irregularly‐orgasmic women, endorsement of OGSJ beliefs was associated with greater sexual satisfaction, even in the absence of regular orgasms, suggesting that OGSJ beliefs offer protection from distress incurred from facing inequality (Study 1). Study 2 showed a descriptively similar pattern using a different operationalization of orgasm consistency; however, the interaction was not significant and should therefore be interpreted with caution. Importantly, irregular orgasm during partnered sex does not necessarily indicate challenges with orgasm functioning, as women often report higher orgasm frequency during masturbation than during partnered sexual activity (Laumann et al., 1994; Rowland, Sullivan, et al., 2018). This suggests that personal experiences which contradict the status quo are insufficient to attenuate system‐justifying beliefs.

Orgasm gap‐system justification (OGSJ) beliefs may make individual women feel better in one sense (increased sexual satisfaction), but they do not improve women's orgasm‐related outcomes. OGSJ could contribute to a palliative trap, keeping women stuck in self‐limiting beliefs about not “needing” orgasms, thereby lowering their expectations (Goldey et al., 2016) and deferring their chances of embodied sexual pleasure (Wetzel et al., 2022). Indeed, we found that higher OGSJ was associated with reduced orgasm centrality and lower feelings of orgasm entitlement for women, whereas for men it was linked to greater orgasm entitlement (Study 1). These findings conceptually align with a lower likelihood of behaving in ways that would rectify the orgasm gap. Higher OGSJ also predicted macro‐level acceptance of inequality among both women and men via reduced support for organizational policies that would promote orgasm equality (Study 4). This corroborates past research showing that legitimizing beliefs hinder system‐challenging collective actions (Osborne et al., 2018).

Feminist work has highlighted potential limitations of emphasizing women's orgasms in discussions of sexual equality, including the risk of increasing pressure on women, overlooking ambivalent or negative orgasm experiences, and narrowing sexual pleasure to orgasm alone (see Chadwick, 2024). Related critiques suggest that orgasm‐focused narratives can sometimes reproduce patriarchal norms, suggesting that women do not “need” orgasms (Cerankowski, 2021). However, analogous patterns in other domains suggest that the presence of rationalizations does not imply that the underlying resource is unimportant. For example, women often provide justificatory accounts that render gendered pay disparities subjectively acceptable (e.g., Connor & Fiske, 2019), yet such rationalizations are not typically interpreted as evidence that income is unimportant. Likewise, although women's earnings may be appropriated or controlled by others in contexts of coercion, this does not lead researchers to conclude that income itself is inherently patriarchal or that women should disengage from pursuing higher wages and salaries.

While we agree that orgasm is only one component of sexual pleasure, our findings (Studies 1 and 2) support extensive existing evidence that orgasming is an important factor in women's sexual well‐being (e.g., Dienberg et al., 2023). A system justification perspective is also consistent with the idea that when women do not expect orgasms, they may redefine sexual satisfaction in compensatory ways, such as emphasizing intimacy or their partner's pleasure (Blumenstock, 2022). Although many aspects of sexuality are meaningful, concern is warranted when pleasurable, rewarding components are distributed or experienced unequally between groups (Chadwick, 2024; McClelland, 2011). We therefore suggest that challenging system‐justifying beliefs about the orgasm gap may expand individuals' freedom to decide whether, how, and how often they wish to experience orgasm, rather than having these expectations shaped by gendered inequalities.

### Motivated maintenance of inequality

The present study package also provides evidence consistent with another main tenet of SJT: that women's OGSJ endorsement reflects a motivated process, expressed through biased information processing. We observed these effects primarily among irregularly‐orgasmic women, who, when given the choice, actively selected information that affirmed the status quo (Study 2). This is consistent with prior findings that disadvantaged group members may selectively expose themselves to information that preserves system legitimacy (Hennes et al., 2016). Irregularly‐orgasmic women further tended to delegitimize system‐threatening information when presented with it (Study 3). This aligns with research showing that individuals react defensively when faced with system‐threatening information (e.g., Brescoll et al., 2013; Laurin et al., 2011, for an overview see Jost, 2019), which may involve discounting or devaluing such information (Ledgerwood et al., 2011). Our findings reflect what motivated reasoning theory describes—people process information in ways that help them reach desired conclusions (Kunda, 1990). Women who do not usually orgasm during partnered sex may be especially motivated to protect the legitimacy of the gendered sexual status quo by either embracing system‐affirming explanations or discounting those that challenge it.

Exploratory analyses hinted towards another SJT‐consistent strategy: denial of personal relevance. Both women and men perceived the distribution of orgasms in their own relationships as relatively fair but acknowledged inequality at the societal level (Study 4). As prior work in SJT literature has found, people distance themselves from systemic injustices when change feels unachievable (Napier et al., 2020; Paulhus, 1984). Together, these results show that OGSJ is neither a passive acceptance of gender inequality nor a mere internalization of gender norms, but rather reflects an active, multi‐faceted effort to maintain psychological and relational stability (Jost et al., 2010).

There are alternative, but related, psychological explanations for women's acceptance of orgasm inequality. For example, cognitive dissonance theory (Festinger, 1957) has been suggested by previous researchers to explain why women might devalue orgasm (Janssen et al., 2025; Laan et al., 2021). If women value orgasm but do not regularly experience it, this discrepancy may create psychological discomfort, which can be reduced by adjusting beliefs about orgasm's importance. In this sense, cognitive dissonance can be understood as one route to ego justification: redefining orgasm as less important may protect the self from feelings of inadequacy or personal failure (Laan & Rellini, 2011).

However, this account has important limitations. Cognitive dissonance theory assumes that belief change is driven by conscious psychological discomfort (Jost, 2019). Yet our findings include relatively high levels of sexual satisfaction among irregularly‐orgasmic women—suggesting little conscious distress with lower orgasm rates—while still engaging in system‐justifying responses when confronted with system‐threatening information. This pattern is difficult to reconcile with a purely dissonance‐based account but is consistent with system justification theory, which does not require conscious distress (Jost & Hunyady, 2003).

In addition, a dissonance‐based account remains focused on ego‐protective processes and does not fully explain why these adjusted beliefs systematically align with gendered norms (e.g., the belief that men's orgasms are more natural or more important). System justification theory, on the other hand, also considers the threats to individual well‐being posed by group‐level and structural‐level dynamics. In the context of the orgasm gap, ego‐protecting and system‐justifying motives may operate in greater alignment for irregularly‐orgasmic women. For women who do not usually orgasm, adopting system‐affirming beliefs (e.g., that women's orgasms are naturally more complex) may simultaneously serve a self‐protective function (“I want to feel adequate”) and a system‐justifying function (“this is natural and expected”), providing a coherent explanation of the status quo.

### Limitations and future directions

There are several limitations to our study. Although OGSJ beliefs appear to serve a palliative function for irregularly‐orgasmic women, not all women in this group endorsed such beliefs. Personality factors such as a high need for cognitive closure or low openness to experience may increase motivation to adopt stable, certainty‐providing explanations, making system‐justifying beliefs more appealing (Jost & Hunyady, 2005). Similarly, relational factors such as perceived partner dependence or relational interdependence may increase motivation to maintain harmony and stability within relationships at the expense of challenging inequality (Sobol‐Sarag et al., 2023). We might especially expect this trade‐off among women in relationships with men, as they may be prone to experience relational interdependence characterized by emotional closeness and a focus on commonalities with their partner (Sobol‐Sarag et al., 2023). Future research should examine whether these personal and relational factors increase the likelihood of legitimizing the orgasm gap, for example by testing whether women with less independence in their relationships are more likely to endorse OGSJ beliefs.

A further limitation is that the proportion of irregularly orgasmic participants in our study was relatively low, especially in Study 3. Sexuality research is subject to self‐selection biases, with more sexually progressive individuals—particularly women—likely overrepresented (e.g., Wiederman, 1999). As such, irregularly‐ or non‐orgasmic women may be underrepresented. Moreover, our data do not allow us to determine why some women report low or no orgasm frequency. Certain physical conditions—such as pelvic trauma, spinal cord injuries, surgical interventions to the vagina or vulva, or hormonal changes during menopause—may limit orgasm capacity (Laan & Rellini, 2011; Stuparu, 2020). However, studies suggest that psychological and relational factors are more commonly implicated in women's low orgasm rates (Laan & Rellini, 2011; Lavie‐Ajayi, 2005; Moura et al., 2020; Rowland, Cempel, & Tempel, 2018), indicating that biological factors represent only one part of a broader, multifactorial phenomenon. Psychological factors, including beliefs about gender and sexuality, may also differ between women with different patterns of orgasm frequency and help explain variation in the tendency to justify the orgasm gap. Importantly, however, the specific causes of low orgasm frequency do not alter the theoretical logic of the present research. Different reasons for women's low orgasm frequency may be associated with varying degrees of OGSJ, but SJT predicts that disadvantaged individuals may rationalize unequal outcomes regardless of whether their position results from biological, psychological, relational, or sociocultural factors (Jost, 2020).

Neither do our data allow us to disentangle the motives underlying OGSJ. According to system justification theory, endorsement may serve epistemic (reducing uncertainty), existential (reducing threat), and relational motives (maintaining harmony and shared reality; Jost et al., 2008; Hardin & Conley, 2001; Hardin & Higgins, 1996). Interpersonal needs for shared reality may be especially relevant in intimate relationships, where justifying beliefs can help preserve harmony and avert conflict. Consistent with this, research on benevolent sexism—closely linked to system justification—shows that women who endorse such beliefs may avoid interpersonal costs, such as social stigma (Glick et al., 2000).

At the same time, our findings that essentialist narratives about sex and gender were associated with OGSJ point to the role of epistemic motives. These narratives implicate dominant sexual scripts that prioritize male pleasure and position women's pleasure as secondary (Sanchez et al., 2012). Future research could examine how exposure to, or internalization of, these beliefs jointly shape expectations and experiences of sexual pleasure, and how they interact over time to maintain or challenge the orgasm gap. Finally, as discussed above, system‐justifying processes may in some cases be accompanied by ego‐justifying motivations, such as reducing feelings of personal inadequacy. Future research is needed to disentangle these potentially overlapping motivational processes and to clarify the relative contributions of epistemic, relational, and self‐protective motives in sustaining a gendered system in mixed‐gender sexual relationships.

In these studies, we provided (to our knowledge) the first evidence that men, like women, engage in OGSJ and that it is linked to reduced interest in social change supporting gender equality. Unfortunately, the motivational strategies and antecedents underlying men's endorsement of OGSJ were beyond the scope of this research. Past research suggests that gender identity salience may play a role: men who are highly identified with their male gender are more likely to engage in system justification (Domen et al., 2022). In contrast, egalitarian values appear to shape more equity‐oriented sexual behaviour, as feminist‐identified men report greater motivation to please their female partners (Stick & Fetner, 2021). Yet, this picture is further complicated by evidence that some men pursue their partner's orgasm not to promote gender equality, but to affirm their own masculinity (Chadwick & van Anders, 2017). Future research is needed to disentangle the social and psychological dynamics that drive men's justification of orgasm inequality and how these motivations may differ across contexts and identities.

One methodological limitation of the present research is the use of a novel measure to assess orgasm gap‐system justification. This decision was necessary because no existing validated scale captured system‐justifying beliefs in the context of gendered inequalities in orgasm rates. We therefore adapted an established measure of system justification of gender income inequality (Connor & Fiske, 2019), modifying minimal item wording to reflect disparities in orgasm frequency (instead of income) while retaining the original structure. The scale showed expected associations with related constructs (e.g., conservatism and gender‐specific system justification), but it has not yet undergone extensive psychometric validation. Accordingly, future studies should further validate this measure.

Another methodological limitation is that Studies 2–4 were not preregistered. Preregistration is, of course, an important tool for increasing transparency and reducing researcher degrees of freedom. However, the present studies were theory‐driven and the analytic strategy was applied consistently across studies. Importantly, direct replication provides a particularly stringent test of the robustness of findings (Roediger, 2012), and the pattern of results was highly consistent across independent samples. Furthermore, additional robustness analyses yielded substantively identical conclusions.

Lastly, support for SJT is not without debate. Critics argue that a distinct system‐justification motive may not be necessary, and that apparent system‐justifying tendencies can instead be explained by social identity processes (Owuamalam et al., 2018, 2019). Specifically, they propose that such responses may reflect (a) superordinate in‐group bias, whereby individuals support the system because they identify with a broader collective, (b) passive acceptance of social reality, in which individuals simply accept the status quo without active motivation, or (c) hope for future group advancement, whereby disadvantaged individuals tolerate inequality in anticipation of future improvement.

However, the present research was designed to assess the rationalization of the gender orgasm gap within the micro‐system context of dyadic sexual relationships. As such, the superordinate in‐group account (a) is less applicable in this context, as we focused on how OGSJ interfaces with individuals' personal orgasm rates, and not on how it may vary with the strength of their gender identity. Future work could assess whether individuals are more motivated to rationalize the orgasm gap to protect their interpersonal, relational well‐being or to protect their feelings of belonging to their gender group. As for (b), a passive acceptance explanation is difficult to reconcile with our findings because participants did not simply accept the status quo but showed active, motivated processing—selectively endorsing system‐affirming explanations of the orgasm gap and discounting system‐threatening information.

Finally, a hope‐for‐future‐advancement account (c) appears unlikely given the broad endorsement of biologically essentialist beliefs across participants in our studies. Characteristics of people that are understood as “essential” tend to be perceived as immutable. Gender is a relatively stable social category, making it unclear why women would justify unequal orgasm outcomes based on expectations of future improvement. Therefore, we find it unlikely that women's present endorsement of OGSJ is only in anticipation of future progress towards orgasm equality. Future research could compare how social identity threats and status quo threats are associated with variance in OGSJ to better elucidate women and men's motivations for tolerating this persistent gender inequality.

## CONCLUSION

Our findings across four studies demonstrate that justification plays an important role in sustaining the orgasm gap: it offers a palliative function for women who do not usually orgasm during sex with men, is driven by biased information processing, and is linked to reduced support for efforts aimed at promoting sexual equality. Together, these findings extend SJT by illustrating how inequality is preserved not just in institutions and policies, but also in intimate spaces.

## AUTHOR CONTRIBUTIONS

**Tanja Oschatz:** Data curation; formal analysis; project administration; writing – original draft; writing – review and editing. **Verena Klein:** Conceptualization; data curation; project administration; writing – original draft; writing – review and editing; methodology; supervision; resources. **Jennifer L. Piemonte:** Formal analysis; writing – original draft; writing – review and editing. **Roland Imhoff:** Writing – review and editing. **Terri Conley:** Conceptualization; methodology; resources; writing – review and editing; supervision; data curation.

## FUNDING INFORMATION

This study was supported by a Ph.D. scholarship of the Friedrich Ebert Foundation awarded to Tanja Oschatz. Verena Klein was supported by a Starting Grant from the European Research Council (ERC), fulfiled through the UKRI [EP/Y032608/1].

## CONFLICTS OF INTEREST STATEMENT

The authors declare that they have no conflict of interest.

## ETHICS STATEMENTS

The studies were exempt from ethical approval by the University of Michigan (HUM00195714).

## CONSENT TO PARTICIPATE

Written informed consent to participate in this study was obtained from all individual participants prior to their inclusion.

## Supporting information


**Data S1:** Supporting Information.

## Data Availability

The data that support the findings of this study are openly available in Open Science Framework at https://osf.io/4afb6/?view_only=78be58f8a5d44a3eb7eca230e90e489d, reference number 4afb6.

## Footnotes

^1^
Please note that the current power calculation differs from the pre‐registered one, as the pre‐registration did not base the estimate on the most complex planned analysis (i.e., the moderation model) but was rather conducted for a simple regression model.

^2^
Although concerns regarding MTurk data quality had already emerged by the time these data were collected (e.g., Chmielewski & Kucker, 2020), several studies have found that with appropriate quality‐control procedures, MTurk data are comparable in quality to more traditional research samples like undergraduate students or online community samples (e.g., Kees et al., 2017; Zhang & Gearhart, 2020). To ensure sufficient data quality, we used brief surveys, excluded ineligible and inconsistent responses according to predefined criteria (as specified in the current Method section). Most importantly, the robustness of our findings across samples provides additional reassurance, since we observed a consistent pattern of results across four independent studies, including one conducted on Prolific.

^3^
We additionally explored the effects of the experimental manipulation on women's orgasm centrality, finding that women exposed to system‐threatening narratives rated orgasm as slightly more central to their sexual satisfaction than those in the system‐affirming condition. The interested reader can find the results on the OSF.

^4^
Our dichotomous item (“generally orgasm”: yes/no) captures whether women usually orgasm, not how often relative to a male partner. Thus, many “yes” responses can coexist with a persistent orgasm gap.

^5^
As a robustness check of our main results, we also examined the dependent variable of plausibility alongside credibility. The pattern of results replicated those reported here and are available on OSF.

^6^
We conducted an additional study with a male sample to examine their role in justifying the OG. For brevity reasons, the full methods, analyses, and results of this study are reported in detail on the OSF.
