# Supplementary material for: How system justification sustains sexual inequality: The case of the orgasm gap
Source: Br J Soc Psychol. 2026 Oct 22;65(4):e70141. doi: 10.1111/bjso.70141 (PMC13687909; doi:10.1111/bjso.70141)
Supplement: Supplementary file 1 [file BJSO-65-0-s001.zip › Study 1/CFA.docx]

**Table 1**

*Confirmatory Factor Analysis*

| **Items** | Standardized Factor Loadings | |
| --- | --- | --- |
|  | 7- Item Model | 6- Item Model |
| 1. Differences in the orgasm frequency between men and women are too large. (R) | .60 | .64 |
| 2. Figuring out how to minimize the orgasm gap between men and women would create more problems than it would solve. | .36 |  |
| 3. Orgasm equality between men and women should be our ideal. (R) | .60 | .62 |
| 4. We should always strive to make men’s and women’s orgasm frequency more equal. (R) | .62 | .65 |
| 5. Differences in orgasm frequency between men and women are justified. | .80 | .75 |
| 6. The current extent of orgasm inequality between men and women in our society is not a serious problem. | .71 | .71 |
| 7. There are perfectly understandable reasons why men and women often experience different frequencies of orgasm. | .63 | .63 |
| **Model Fit Indices** |  |  |
| CFI | .884 | .933 |
| SRMR | .088 | .076 |
| RMSEA | .172 | .160. |

The confirmatory factor analysis was conducted using *AMOS 27* (Finch et al., 2016), employing maximum likelihood estimation procedures. Model fit was evaluated using multiple indices, including the comparative fit index (CFI) and the standardized root mean square residual (SRMR), following common recommendations in the SEM literature (Hu & Bentler, 1999). We prioritized these indices over the χ² goodness-of-fit test, as χ² is highly sensitive to sample size and inter-item correlations (Hooper et al., 2008). We gave a preference to these indices over the *χ*^2^ goodness-of-fit test, as *χ*^2^ tests are heavily dependent on sample size and correlations among variables (e.g.: Hooper et al., 2008). Fit can be considered acceptable if CFI is .90 or greater and SRMR values are .10 or lower (Hu & Bentler, 1998; Kenny, 2020). For the 7-item model, fit indices indicated insufficient fit for CFI (CFI = .884), and an acceptable fit for SRMR (SRMR = .088). The 6-item model showed improved fit, reaching acceptable levels for both indices (CFI = .933, SRMR = .076), alongside consistently moderate to strong standardized factor loadings (.62–.75).

The root-mean-square error of approximation (RMSEA) scores were above the threshold of .10. Please note that RMSEA scores should not be interpreted in the present analyses. As argued by Kenny and colleagues (2014), the RMSEA is problematic and potentially misleading in models with small degrees of freedom, as it tends to overestimate model misfit even when the model is correctly specified. They explicitly recommend avoiding the use of RMSEA in such cases. Given the relatively low degrees of freedom in the current models, we therefore do not consider the RMSEA values informative for evaluating model fit.

References

Finch, H. W., Immekus, J. C., & French, B. F. (2016). *Applied psychometrics using SPSS and AMOS.* Information Age Publishing Inc.

Hooper, D., Coughlan, J., & Mullen, M. (2008). Structural equation modelling: Guidelines for determining model fit. *Electronic Journal of Business Research Methods, 6*, 53–60.

Hu, L. T., & Bentler, P. M. (1998). Fit indices in covariance structure modeling: Sensitivity to underparameterized model misspecification. *Psychological Methods, 3*(4), 424-453. <https://psycnet.apa.org/doi/10.1037/1082-989X.3.4.424>

Kenny, D. A. (2020). *Measuring model fit*. http://davidakenny.net/cm/fit.htm

Kenny, D. A., Kaniskan, B., & McCoach, D. B. (2015). The performance of RMSEA in models with small degrees of freedom. *Sociological methods & research*, *44*(3), 486-507. <https://doi.org/10.1177/0049124114543236>
