# Supplementary material for: How system justification sustains sexual inequality: The case of the orgasm gap
Source: Br J Soc Psychol. 2026 Oct 22;65(4):e70141. doi: 10.1111/bjso.70141 (PMC13687909; doi:10.1111/bjso.70141)
Supplement: Supplementary file 1 [file BJSO-65-0-s001.zip › Study 1/Additonal Results_Study 1.docx]

| **Outcome** | ***R^2^*** | ***F*** | ***p*** | **Predictors** | | |
| --- | --- | --- | --- | --- | --- | --- |
|  |  |  |  |  | ***ß*** | ***p*** |
| Orgasm Centrality | .04 | 3.72 | .012 | OGSJ | -.17 | .008 |
|  |  |  |  | Relationship Status | -.10 | .107 |
|  |  |  |  | Age | .04 | .546 |
| Orgasm Entitlement | .09 | 7.95 | <.001 | OGSJ | -.27 | <.001 |
|  |  |  |  | Relationship Status | -.11 | .069 |
|  |  |  |  | Age | .02 | .713 |

**Table 1**

*Regression Results for Influence of OGSP, Relationship Stauts and Age on Women’s Orgasm Centrality and Entilement*

*Note.* OGSJ = Orgasm Gap System Justification; Relationship Status was coded “Single” =1 and “In a relationship” = 2.

| **Outcome** | ***R^2^*** | ***F*** | ***p*** | **Predictors** | | |
| --- | --- | --- | --- | --- | --- | --- |
|  |  |  |  |  | ***ß*** | ***p*** |
| Orgasm Entitlement | .11 | 12.05 | <.001 | OGSJ | .31 | <.001 |
|  |  |  |  | Relationship Status | -.04 | .419 |
|  |  |  |  | Age | .09 | .119 |

**Table 2**

*Regression Results for Influence of OGSP, Relationship Stauts and Age on Men’s Orgasm Centrality and Entilement*

*Note.* OGSJ = Orgasm Gap System Justification; Relationship Status was coded “Single” =1 and “In a relationship” = 2.
