# Supplementary material for: How system justification sustains sexual inequality: The case of the orgasm gap
Source: Br J Soc Psychol. 2026 Oct 22;65(4):e70141. doi: 10.1111/bjso.70141 (PMC13687909; doi:10.1111/bjso.70141)

Adiditonal Results

Study 3

Method

Plausibility

Participants were then asked: "*How much do you believe the presented research results?"*. The item was answered on a 6-point scale ranging (1= *not at all* and 6 = *completely*).

Orgasm Centrality

Orgasm centrality was assessed as in Study 1 (Cronbach's α = .85).

Results

Plausibility

Results revealed a significant main effect of condition on plausibility, $F(1, 325) = 61.99$, $p < .001$, $\eta_p^2 = .160$, such that participants in the affirming condition ($M = 4.93$, $SD = 1.15$) reported higher belief in the research than participants in the threatening condition ($M = 3.75$, $SD = 1.75$), $t(327) = 7.19$, $p < .001$, $d = 0.79$, 95% CI [0.86, 1.51]. Additionally, there was a significant main effect of orgasm presence on plausibility, $F(1, 325) = 11.39$, $p < .001$, $\eta_p^2 = .034$. Participants with a high orgasm rate ($M = 4.51$, $SD = 1.50$) reported higher beliefs in the research compared to participants with a low orgasm rate ($M = 3.80$, $SD = 1.77$), $t(327) = 3.67$, $p < .001$; $d = 0.45$, 95% CI [0.33, 1.10].

Again, the predicted interaction between condition and orgasm presence was significant, $F(1, 325) = 9.90$, $p = .002$, $\eta_p^2 = .030$. In the system-affirming condition, there was no difference in the plausibility item between women with a low orgasm rate ($M = 4.90$, $SD =$

1.15) or a high orgasm rate (*M* = 4.94, *SD* = 1.15), *t*(157) = 0.20, *p* = .423, *d* = 0.04, 95% CI [-0.38, 0.46]. In contrast, in the system-threatening condition, women with a high orgasm rate (*M* = 4.09, *SD* = 1.67) judged the research to be significantly more believable compared to women with a low orgasm rate (*M* = 2.92, *SD* = 1.67), *t*(168) = 4.15, *p* < .001, *d* = 0.70, 95% CI [.62, 1.73].

Orgasm Centrality

Regarding orgasm centrality there was a significant main effect of condition, *F*(1, 325) = 7.83, *p* = .005, ηp^2 = .024, such that participants in the system-threatening condition reported slightly higher orgasm centrality than those in the system-affirming condition. There was also a large main effect of orgasm consistency, *F*(1, 325) = 83.33, *p* < .001, ηp^2 = .204, with consistently-orgasmic women reporting greater orgasm centrality than inconsistently-orgasmic women. However, the interaction between condition and orgasm consistency was not significant, *F*(1, 325) = 0.18, *p* = .674, ηp^2 = .001. Please see Figure 5.

Discussion

Results for credibility were replicated using the variable of plausibility. Additionally, we found a small but significant effect of condition on orgasm centrality: women exposed to system-threatening narratives rated orgasm as slightly more central to their sexual satisfaction than those in the affirming condition. This suggests that system-justifying narratives surrounding the female orgasm are not only pervasive but also psychologically impactful—even with brief exposure and even among women who regularly experience orgasm.

Figure 5

Interaction Between Condition and Orgasm Consistency on Orgasm Centrality

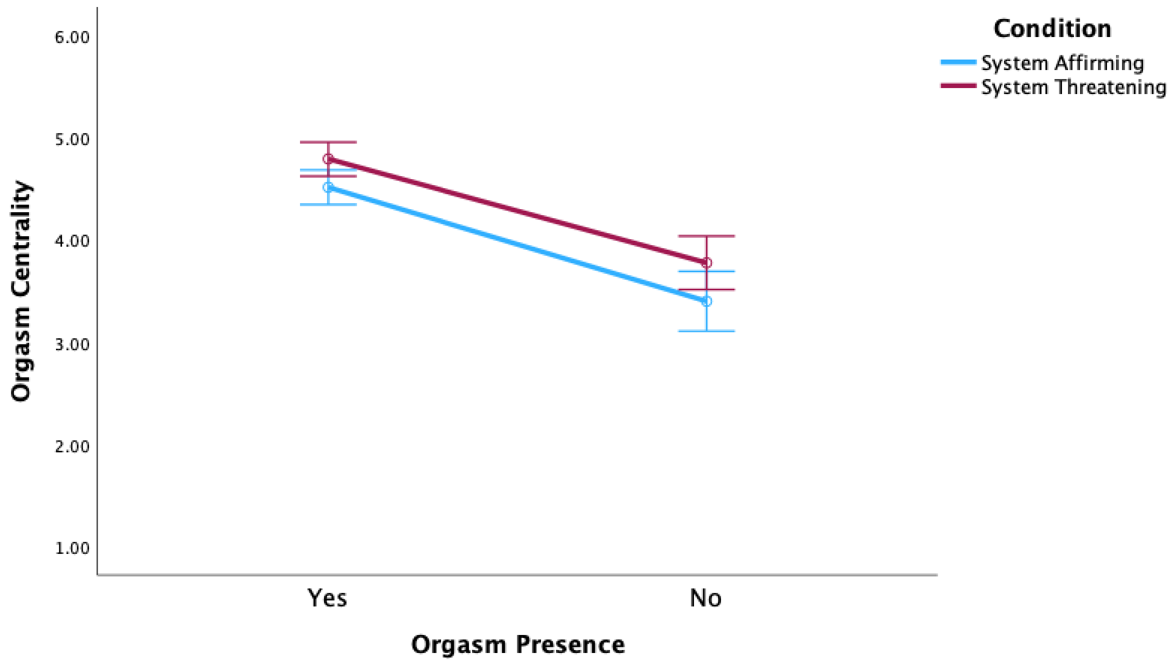

Supplement: Supplementary file 1 [file BJSO-65-0-s001.zip › Study 3/Adiditonal Results_Study 3.pdf]
