# Supplementary material for: How system justification sustains sexual inequality: The case of the orgasm gap
Source: Br J Soc Psychol. 2026 Oct 22;65(4):e70141. doi: 10.1111/bjso.70141 (PMC13687909; doi:10.1111/bjso.70141)
Supplement: Supplementary file 1 [file BJSO-65-0-s001.zip › Additional Material and Data/Additional Results_Study Men.pdf]

Study 5

We aimed to test the motivational underpinnings of men's responses to gender-based sexual inequality. We examined whether threatening the status-quo (here, the orgasm gap that advantages men) increases men's tendencies to justify gender inequality in their own sexual relationships.

We hypothesized that, compared to a control condition, men exposed to a system threat would be more motivated to justify the pleasure distribution within their own relationships. We focused on system justification within participants' personal relationships, drawing on research showing that threats to large-scale system (the societal gendered orgasm gap) can lead to bolstering the legitimacy of small-scale systems (one's personal relationship; Wakslak et al., 2011). Building on prior work showing that gender threats elicit stronger reactions among men highly identified with being male (Domen et al., 2022), we also tested whether this effect would be moderated by male gender identification. This study was pre-registered: https://aspredicted.org/dqjv-zbns.pdf.

Exploratively, we aimed to investigate how men perceive the fairness of the OG at both the intrapersonal (within their own relationships) and the interpersonal or societal level.

Method

Participants

An a priori power-analysis for a one-tailed independent-sample t-test ($d = 0.4$, 80% power, $\alpha = .05$) indicated that 156 participants would be needed. We collected a total sample of 238 participants. We excluded 20 participants due to incomplete data sets, 10 due to a failed attention check, and one person because they identified as homosexual. This resulted in a final sample of 207 participants ($M_{\text{age}} = 39.75$, $SD_{age} = 12.19$, range = 18-76).

Table 1

Sociodemographic Characteristics of Participants

	Study 4	
	N	%
Gender		
Women	0	0
Men	207	100
Sexual orientation		
Heterosexual	195	94.2
Bisexual	12	5.8
Ethnicity		
African American/Black British/Black	52	25.1
Asian American//Asian	15	7.2
European American/White	123	59.4
Hispanic/Latina	13	6.3
Middle Eastern	0	0
Native/American Indian	2	1.0
Multi-ethnoracial	2	1.0
Education		
Less than high school	1	0.5
High school diploma	35	16.9
2-year college/university degree	20	9.7
4-year college/university degree	76	36.7
Master’s degree	55	26.6
Professional degree (JD, MD)	5	2.4
Doctoral degree	15	7.2
Relationhsip status		
Single	196	94.7
In Relationship	11	5.3

Procedure

After providing informed consent, participants were randomly assigned to one of two conditions (experimental vs. control). As part of the cover story to keep the research purpose concealed, the study was described as exploring how different types of audiovisual content – specifically informative podcasts – impact people's personal perspectives and engagement with social topics. We scripted and developed fictional podcast segments to serve as the present study's experimental stimuli. All participants were presented with a one-minute video clip, described as a segment from a recent podcast episode, and featured a female voice narrating over an animated video displaying related graphics. Following the podcast segment, participants completed a manipulation check, variable measures, demographic questions, and an attention check before receiving debriefing information about the faux podcast.

Manipulation of System Threat

In the system threat condition, participants listened to a segment of a "podcast episode" hosted by a fictitious expert, Dr. Lucy Holmes, called "The Sexpert." She defines the orgasm gap and describes it as a gender-based inequality that benefits men, how it cannot be explained by biological differences, and that to achieve equality, men should be prepared to reduce their own orgasm frequencies. The segment described an (also fictitious) active government initiative, *The Orgasm Equality Project*, aimed at closing the gap within the next ten years through measures such as implementing sex education modules that center female pleasure in schools and developing pharmaceutical interventions to reduce male orgasm frequency. The segment also referenced actual women's movements that advocate for sexual withholding as a strategy to pursue women's rights (e.g., strikeforpleasure.com).

In the control condition, participants were exposed to a "podcast excerpt" of equal length on an unrelated, neutral topic called "The Foodie." This segment described a "dessert

gap," where shorter people receive a decreased dessert selection when dining out at restaurants as compared to taller individuals. The stimuli for both conditions were narrated by the same individual, contained almost identical graphics, and had equivalent word counts.

Measures

Manipulation Check. To check the manipulation's effect, we tested whether the threat condition elicited more negative emotions than the control condition among participants with the following items: "How annoying do you find the woman hosting the podcast?" and "How annoying will other people find the woman hosting the podcast?" The scale ranged from 1 (not at all annoying) to 5 (extremely annoying).

System Justification of Personal Sex Life. We measured system justification tendencies by adapting four items from Wakslak and colleagues (2011)'s family system justification scale (e.g., "In general, the sex with your partner is fair and balanced."). The items were measured on a nine-point Likert scale (1 = strongly disagree, 9 = strongly agree). Cronbach's α for this scale was .77.

Self-reported Orgasm Gap in Own Relationship. To assess participants' perceptions of the OG within their own relationships, we asked them to respond to two items: "Please indicate how often the following occur during sexual encounters with your partner: (1) I have an orgasm" and "(2) My partner has an orgasm." Responses were recorded on a five-point Likert scale (1 = Never, 5 = Always). From these two items, we calculated an OG score by subtracting the partner's orgasm frequency from the participant's own. A score of 0 indicates no perceived OG, negative scores reflect a gap favoring the partner (i.e., the partner orgasms more frequently), and positive scores indicate a gap favoring the participant (i.e., the participant orgasms more frequently than their partner).

Fairness Perception Interpersonal. To measure the extent to which participants are aware of pleasure inequality on a societal level we asked participants to rate their agreement with the following item: "The distribution of sexual pleasure experienced by men and women is fair." The item was answered on a seven-point Likert scale (1 = strongly disagree, 7 = strongly agree).

Fairness Perception in Intrapersonal. To assess participant's perception of pleasure distribution within their own relationships they were asked to rate the following item: "The distribution of sexual pleasure between me and by partner is fair." This statement was rated on a seven-point Likert scale (1 = strongly disagree, 7 = strongly agree).

Male Gender Identification. We measured the identification with being a man with five items from Glick and colleagues (2015). An example item is: "Being a man is an important part of who I am." Items were evaluated on a seven-point Likert scale (1 = totally disagree, 7 = totally agree), and the scale showed a Cronbach's α of .90.

Results

Main Analysis

Based on the manipulation check, participants in the threat condition did not judge the podcast host as significantly more annoying (M = 2.22, SD = 1.40) than the control condition (M = 2.02, SD = 1.31), $t(205) = 1.04$, $p = .149$, $d = 0.15$, nor to the perception that other people would judge the podcast host as more annoying (M_{Threat} = 2.58, SD_{Threat} = 1.41; $M_{Control}$ = 2.36, $SD_{Control}$ = 1.30), $t(205) = 1.22$, $p = .133$, $d = 0.16$).

To test our main hypothesis, we conducted an independent-samples t-test comparing men's system justification in their personal sexual relationships between the threat and control conditions. Contrary to expectations, men in the system threat condition (M = 5.41,

SD = 1.01) did not report significantly higher levels of system justification than men in the control condition (*M* = 5.59, *SD* = 1.03), *t*(205) = -1.24, *p* = .108 (two-tailed), *d* = 0.17.

To test whether the effect of system threat on relationship system justification was moderated by male gender identification, we conducted a moderation analysis again using the PROCESS-macro (Hayes, 2018). The overall model was not significant, *F*(3, 203) = 1.72, *p* = .164, accounting for 2.5% of the variance (R^2 = .025). The interaction between condition and male gender identification was not statistically significant, *b* = 0.14, *SE* = 0.11, *t(203)* = 1.29, *p* = .198, 95% CI [–0.08, 0.36]. This indicates that male gender identification did not significantly moderate the effect of the experimental manipulation on system justification.

Explorative Analysis

Correlative results can be found in Table 1. Perceived fairness at the interpersonal and interpersonal levels were positively correlated. Male gender identification was moderately associated with higher perceptions of fairness in both domains, while self-reported OG was not significantly related to any of the other variables. Notably, the overall self-reported OG in the sample was relatively small (*M* = 0.12, *SD* = 0.89), with 55% reporting no difference between their own and their partner's orgasm frequency.

Although men generally perceived the distribution of pleasure within their own relationships as relatively fair (*M* = 5.80, *SD* = 1.26), they rated the broader societal distribution of sexual pleasure between women and men as significantly less fair (*M* = 4.71, *SD* = 1.58), *t*(205) = 9.95, *p* <.001, *d* = 0.69. Lastly, male gender identification was significantly associated with both OGSJ on a societal level, as well as with the level of fairness attributed to own personal sexual relationship.

Table 1

Variable Correlations by Participants Gender

Variable	1	2	3	4
1. Perceived OG	—			
2 Fairness in Perception Interpersonal	-.01	—		
3 Fairness Perception Intrapersonal	-.02	.395**	—	
4 Male Gender Identification	-.04	.294**	.138*	—
M, SD	0.12 (0.89)	4.71 (1.58)	5.80 (1.26)	5.46 (1.28)

Note. $^{**}p < .01$ $^{*}p < .05$

Discussion

Contrary to expectations, we found no significant effect of the system threat manipulation on men's reports of fairness, satisfaction, or balance in their personal sex lives. In fact, the pattern of results was in the opposite direction: men in the threat condition reported slightly lower levels of system justification, though not significantly so.

These findings suggest that the manipulation may not have effectively induced a sense of system threat. The manipulation check showed no significant differences in participants' perceptions of the podcast host's annoyance, indicating that the message may have been interpreted as informative rather than confrontational.

It is also possible that the broader political context affected the manipulation's plausibility. The study was conducted in December 2024, shortly after the re-election of Donald Trump. In this political climate, the idea of a governmental campaign aimed at promoting sexual equality may have seemed implausible to participants, thereby weakening the intended threat.

Looking at the exploratory results, Study 4 showed that many men do not perceive the OG as a personal issue. Over half of the participants reported no OG in their own relationships. This aligns with dyadic data showing that men systematically underestimate the OG within their own relationships (Wetzel et al., 2022). Moreover, most men rated the distribution of sexual pleasure with their partner as fair—even while acknowledging that inequality exists at the societal level. This tendency to view one's own situation as fair, while recognizing broader injustice, may reflect a subtle form of system justification, in the form of denial (Napier et al., 2020). In such cases, advantaged group members uphold the legitimacy of the system by psychologically distancing themselves from its inequities (Jost & Hunyady, 2002; Wakslak et al., 2011). Similar patterns have been observed in other domains: when confronted with evidence of racial inequality, members of advantaged racial groups often deny their own privilege while still acknowledging systemic disparities (Phillips & Lowery, 2015).
