# Supplementary material for: How system justification sustains sexual inequality: The case of the orgasm gap
Source: Br J Soc Psychol. 2026 Oct 22;65(4):e70141. doi: 10.1111/bjso.70141 (PMC13687909; doi:10.1111/bjso.70141)
Supplement: Supplementary file 1 [file BJSO-65-0-s001.zip › Additional Material and Data/Study Protocol.docx]

**Orgasm gap-system justification** (adapted from Connor & Fiske, 2019)

1. Differences in the orgasm frequency between men and women are too large. (R)
2. Figuring out how to minimize the orgasm gap between men and women would create more problems than it would solve.*
3. Orgasm equality between men and women should be our ideal. (R)
4. We should always strive to make men’s and women’s orgasm frequency more equal. (R)
5. Differences in orgasm frequency between men and women are justified.
6. The current extent of orgasm inequality between men and women in our society is not a serious problem.
7. There are perfectly understandable reasons why men and women often experience different frequencies of orgasm.

** Item was excluded from analysis due to poor fit.*

**Gender-Specific System Justification** (Jost & Kay, 2005)

1. In general, relations between men and women are fair.
2. The division of labor in families generally operates as it should.
3. Gender roles need to be radically restructured. (R)
4. For women, the United States is the best country in the world to live in.
5. Most policies relating to gender and the sexual division of labor serve the greater good.
6. Everyone (male or female) has a fair shot and wealth and happiness.
7. Sexism in society is getting worse every year. (R)
8. Society is set up so that men and women usually get what they deserve.

**Orgasm Centrality** (Rubin et al., 2019)

1. I expected orgasm.
2. Orgasming was a priority for me.
3. I was interested in orgasming.
4. Orgasming was important for my sexual satisfaction.

**Orgasm Entitlement** (adapted from Klein & Conley, 2021; Klein et al., 2024)

1. Generally speaking, during a sexual encounter whose orgasm is more important?
2. Generally speaking, during a sexual encounter, whose orgasm is more necessary?
3. … who should have an orgasm? *(1= Me, 8 = My Partner)*
4. Please indicate whose orgasm was more important during your most recent sexual encounter.

**Support for Gender Equality** (Becker & Wright, 2011)

1. I would participate in a rally demanding equal salaries for women and men.
2. I would sign an online petition to establish a law demanding minimum quotas for women in leading positions.
3. I would act against sexism in general.

**Sources:**

Becker, J. C., & Wright, S. C. (2011). Yet another dark side of chivalry: Benevolent sexism undermines and hostile sexism motivates collective action for social change. *Journal of Personality and Social Psychology*, *101*(1), 62-77. <https://doi.org/10.1037/a0022615>

Connor, R. A., & Fiske, S. T. (2019). Not minding the gap: How hostile sexism encourages choice explanations for the gender income gap. *Psychology of Women Quarterly*, *43*(1), 22-36. <https://doi.org/10.1177/0361684318815468>

Jost, J. T., & Kay, A. C. (2005). Exposure to benevolent sexism and complementary gender stereotypes: consequences for specific and diffuse forms of system justification. *Journal of Personality and Social Psychology*, *88*(3), 498-509. <https://doi.org/10.1037/0022-3514.88.3.498>

Klein, V., & Conley, T. D. (2021). The role of gendered entitlement in understanding inequality in the bedroom. *Social Psychological and Personality Science, 13*(6), 1047-1057. <https://doi.org/10.1177/19485506211053564>

Klein, V., Kosman, E., & Kahalon, R. (2024). Devaluation of women’s sexual pleasure: Role of relationship context and endorsement of the Madonna-Whore Dichotomy. *Sex Roles, 90*(1), 67-81. <https://doi.org/10.1007/s11199-023-01424-3>

Rubin, J. D., Conley, T. D., Klein, V., Liu, J., Lehane, C. M., & Dammeyer, J. (2019). A cross-national examination of sexual desire: The roles of ‘gendered cultural scripts’ and ‘sexual pleasure’in predicting heterosexual women's desire for sex. *Personality and Individual Differences*, *151*, 109502. <https://doi.org/10.1016/j.paid.2019.07.012>
